# A Multi-Layered Proteogenomic Framework for the Prioritization of Cell Surface Therapeutic Targets: Proof-of-Concept for Metastatic Colorectal Cancer

**DOI:** 10.3390/cancers18162570

**Published:** 2026-08-10

**Authors:** Jostein Dahle, Sebastian Patzke

**Affiliations:** 1 Ahus Cancer Research Center, Akershus University Hospital, 1478 Lørenskog, Norway; 2 bioMérieux Norge, Hoffsveien 21, 0275 Oslo, Norway

**Keywords:** proteogenomics, target discovery, cell surface proteins, therapeutic targets, colorectal cancer, metastatic colorectal cancer, target prioritization

## Abstract

Background: Identification of tumor-specific cell surface targets is a critical step in the development of precision oncology therapeutics, including radioligand- and antibody-based approaches. However, existing strategies often rely on single-layer analyses and lack systematic integration of proteomic, genomic, and clinical metadata. Methods: We developed a multi-layered proteogenomic filtering framework integrating quantitative proteomics from colorectal cancer (CRC) cohorts with curated metadata on protein localization, normal tissue expression, and druggability. Eleven complementary filtering strategies were applied, followed by manual curation for extracellular accessibility and composite scoring based on protein rank, localization, and clinical relevance. Results: Application of the pipeline to metastatic CRC (mCRC) identified multiple high-confidence candidate targets, including GPRC5A, SLC2A1, CD47, DPEP1 and IFITM1. The average pairwise overlap between filtering strategies was low (0.11), indicating limited redundancy and complementary target identification across approaches. Importantly, candidates detected by multiple strategies were significantly enriched for established biomarkers (FAP, CEACAM5, ITGAV, ITGB4), which were exclusively found among multi-strategy candidates (10.3% vs. 0%; Fisher’s exact test, *p* = 0.0064), supporting overlap-based prioritization as a marker of biological and translational relevance. Composite scoring further prioritized GPRC5A as a leading candidate. Additional validation layers supported tumor-enriched expression, plasma membrane localization, and relevance across multiple cancer indications. Conclusions: This study presents a scalable framework for prioritization of cell surface therapeutic targets, using mCRC as proof-of-concept indication. By integrating multiple data layers and incorporating translational criteria early in the discovery process, this approach may facilitate more efficient identification of targets for downstream development, including antibody- and radioligand-based therapies.

## Simple Summary

Identifying reliable targets on the surface of cancer cells is essential to developing more precise and effective therapies, but current approaches often rely on limited data and may overlook important factors such as protein location and safety. In this study, we present a new method that combines multiple types of biological and clinical data to systematically identify and prioritize such targets in colorectal cancer. By applying several complementary filtering strategies and focusing on proteins consistently detected across methods, we are able to highlight both well-known and previously underexplored candidates. This approach improves confidence in target selection and reduces the risk of pursuing unsuitable candidates. The framework is flexible and can be applied to other cancer types, helping researchers more efficiently identify promising targets for new therapies and supporting progress in precision oncology.

## 1. Introduction

Target selection is a critical and rate-limiting step in the development of precision oncology therapeutics, particularly for modalities that rely on target accessibility at the cell surface, such as radioligand therapy (RLT), radioimmunotherapy (RIT), antibody-drug conjugates, and other antibody-based platforms [1,2]. The clinical success of these approaches depends on the identification of tumor-associated antigens that are abundantly expressed on the plasma membrane of cancer cells, exhibit limited expression in normal tissues, and present extracellular epitopes amenable to therapeutic binding and internalization. Failure to adequately address these criteria during early target discovery frequently leads to suboptimal efficacy or unacceptable off-target toxicity in later stages of development.

Despite progress in the systemic treatment of metastatic colorectal cancer (mCRC), there remains a substantial unmet need for new therapeutic targets, particularly in patients with refractory or metastatic disease [3]. Current molecularly guided approaches, including anti-EGFR therapy, anti-VEGF therapy, HER2-directed treatment, and immune checkpoint inhibition, benefit selected patient subgroups but do not provide broadly applicable therapeutic options. For surface-directed modalities such as radioligand therapy, radioimmunotherapy, peptide–drug and/or antibody–drug conjugates, bispecific antibodies, and targeted molecular imaging, target selection is especially challenging because clinically useful antigens must combine tumor-enriched expression, plasma membrane localization, extracellular accessibility, limited expression in critical normal tissues, and preferably stable expression across metastatic lesions [4,5]. Many candidates identified through transcriptomic or literature-driven approaches fail to meet these translational requirements. This highlights the need for systematic proteogenomic frameworks that integrate protein abundance, localization, normal tissue expression, and drug development evidence early in the discovery process.

Despite major advances in genomics, transcriptomics, and proteomics, the systematic identification of clinically actionable cell surface targets in solid tumors remains challenging. Many discovery efforts rely predominantly on differential gene expression analyses or literature-driven prioritization, which do not reliably capture protein-level abundance, subcellular localization, or therapeutic accessibility [6,7]. In addition, transcriptomic signals often correlate poorly with surface protein expression, particularly for membrane proteins subject to post-transcriptional regulation, variable trafficking, or proteolytic processing [4,6]. As a result, candidate lists derived from single-layer analyses are frequently enriched for false positives and lack translational robustness.

From a therapeutic standpoint, the “ideal” cell surface target is rarely defined by a single parameter [1,8]. Instead, clinically viable targets reflect a balance between multiple, sometimes competing, criteria, including tumor-selective overexpression, restricted normal tissue distribution, stable membrane localization without extensive shedding, evidence of functional relevance in cancer biology, and feasibility for drug development. Importantly, translational considerations such as prior clinical validation, availability of biomarker data, and compatibility with specific therapeutic modalities are often evaluated late in the discovery process, increasing the risk of downstream attrition.

To address these challenges, there is a growing need for integrative discovery frameworks that combine quantitative proteomics with curated biological and clinical metadata, while explicitly accounting for uncertainty and dataset-specific variability [9,10]. Rather than relying on a single optimal ranking scheme, parallel filtering strategies can provide complementary perspectives on the data, enabling identification of candidates that are robust to parameter selection and cohort effects. Recurrent detection across independent strategies may therefore serve as an implicit robustness metric for target prioritization.

Recent pan-cancer proteogenomic studies have demonstrated the power of integrating tumor-level protein expression with genomic data to expand the landscape of candidate therapeutic targets. In particular, Savage et al. reported a large-scale analysis across multiple cancer types, establishing proteomics as a critical layer for target discovery beyond genomics and transcriptomics alone [9]. However, global target catalogs are not designed to address key translational constraints relevant to therapeutic modalities that require extracellular target accessibility, nor do they provide indication-specific prioritization or robustness assessment under alternative analytical assumptions. These considerations are particularly important for antibody-based therapies and radioligand therapy, where plasma membrane localization and normal tissue expression critically influence clinical feasibility.

Here, we present a multi-layered proteogenomic filtering pipeline designed to systematically identify and prioritize cell surface proteins for therapeutic targeting in solid tumors. The pipeline integrates quantitative proteomics from large-scale cancer cohorts with curated metadata on protein localization, normal tissue expression, expression in cancer cell lines, and clinical drugability. Eleven complementary filtering strategies were applied, followed by manual curation to confirm extracellular accessibility and composite scoring based on expression, localization, and translational relevance. Importantly, the approach emphasizes overlap and consistency across strategies rather than reliance on any single filtering criterion.

We hypothesized that therapeutically relevant cell surface targets in metastatic colorectal cancer (mCRC) could be more robustly prioritized by integrating multiple complementary evidence layers rather than relying on differential expression alone. Specifically, we reasoned that candidate targets supported by tumor-level protein expression, plasma membrane localization, extracellular accessibility, limited normal tissue expression, cancer cell-line expression, clinical or biomarker evidence, and recurrence across independent filtering strategies would be more suitable for downstream surface-directed therapeutic or imaging development [4,5].

mCRC was selected as the proof-of-concept indication because it represents a clinically important disease with substantial unmet need for new therapeutic targets, particularly targets expressed across broader patient populations rather than only small molecularly defined subgroups [3]. This is especially relevant for patients with RAS mutant mCRC, who constitute a large proportion of the mCRC population and have more limited targeted treatment options [11]. Furthermore, mCRC is a clinically relevant setting for the development of surface-directed modalities, including radioimmunoconjugates, radioligand therapy, antibody-based therapies, antibody–drug conjugates, and molecular imaging [4,5].

The availability of large-scale CPTAC colorectal cancer proteomic datasets, together with complementary resources for normal tissue expression, protein localization, cancer cell line expression, and drug development annotation, also made mCRC a suitable data-rich indication for evaluating the proposed prioritization framework [6,7].

The novelty of the present framework lies in its explicit integration of target discovery and translational development criteria within a single prioritization workflow. Unlike approaches that primarily generate broad proteogenomic target catalogues or rank candidates based on differential expression alone, this pipeline focuses specifically on therapeutic accessibility at the cell surface [4,9]. It combines protein-level expression, plasma membrane localization, extracellular accessibility, normal tissue expression, cancer cell line expression, and clinical druggability annotations [1,10]. In addition, the use of multiple complementary filtering strategies allows candidates to be assessed under different analytical assumptions, with recurrence across strategies serving as a practical robustness metric.

We apply this framework to metastatic colorectal cancer (mCRC) as a case study, a disease with high unmet clinical need and increasing interest in targeted radiopharmaceutical and antibody-based therapies. Using data from the Clinical Proteomic Tumor Analysis Consortium (CPTAC) [6,7], Human Protein Atlas [12], Genotype-Tissue Expression project, Cancer Cell Line Encyclopedia, and curated drug development databases, we demonstrate that this multi-layered approach identifies both established colorectal cancer biomarkers and previously underexplored candidate targets. The resulting prioritization highlights GPRC5A as a top candidate while maintaining sensitivity to diverse protein classes and biological mechanisms.

Together, this study provides a scalable and generalizable framework for cell surface target discovery that explicitly integrates proteomic evidence with translational constraints. Beyond colorectal cancer, the modular design of the pipeline enables adaptation to other solid tumor indications and therapeutic modalities, supporting early-stage decision-making in precision oncology drug development. By applying this framework to mCRC, we aimed to address a clinically relevant target discovery problem in a disease where improved surface-directed therapeutic strategies are urgently needed.

## 2. Materials and Methods

### 2.1. Data Sources

The pipeline integrates multiple publicly available and proprietary datasets to enable comprehensive target discovery. Quantitative protein expression data were obtained from the Clinical Proteomic Tumor Analysis Consortium (CPTAC) COAD and COADREAD cohorts, comprising colorectal adenocarcinoma and rectal cancer samples with matched normal tissue controls [6,7]. Protein abundance in normal tissues was assessed using data from the Human Protein Atlas (HPA) and the Genotype-Tissue Expression (GTEx) project [12,13]. RNA sequencing data for colorectal cancer cell lines were retrieved from the Cancer Cell Line Encyclopedia (DepMap) [14]. Clinical metadata describing drugability and biomarker status were sourced from Clarivate Drug Discovery Intelligence (CDDI, Cortellis platform; Clarivate Analytics), while information on the number of unique drugs, associated indications, and highest clinical development stage was extracted from SwissProt [15].

### 2.2. Pipeline Architecture

**Figure 1:**
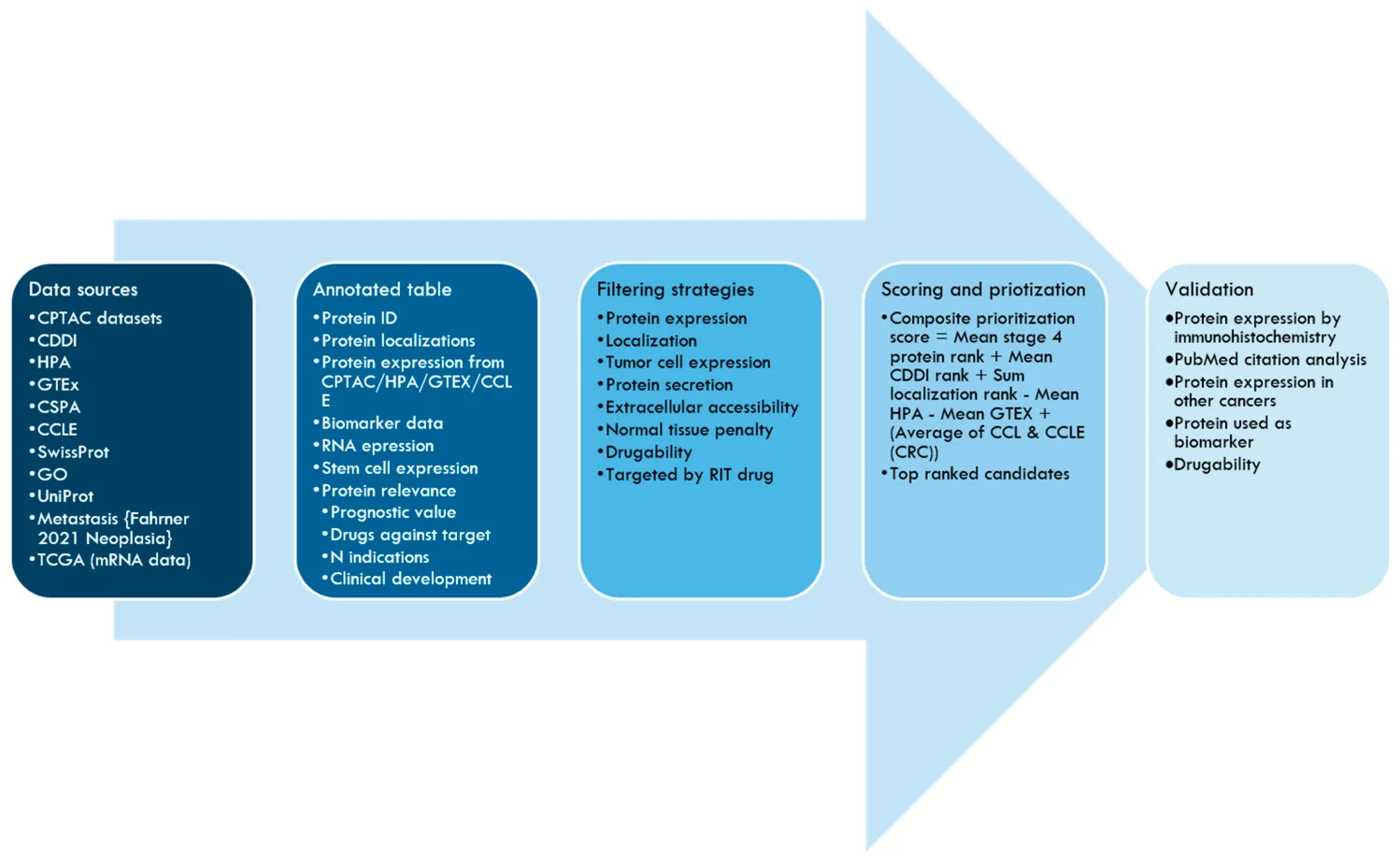
Architecture and workflow of the multi layered proteogenomic filtering pipeline for cell surface target discovery. The pipeline integrates quantitative proteomic data from CPTAC colorectal cancer cohorts with curated biological and clinical metadata to enable systematic prioritization of cell surface therapeutic targets. Multi source datasets, including CPTAC COAD and COADREAD proteomics, Human Protein Atlas (HPA), Genotype Tissue Expression (GTEx), Cancer Cell Line Encyclopedia (DepMap), UniProt, Cell Surface Protein Atlas (CSPA), and Clarivate Drug Discovery Intelligence (CDDI), are merged into an annotated union table containing protein expression metrics, subcellular localization annotations, normal tissue expression levels, tumor cell expression, and drugability information.

The target discovery pipeline was designed as a modular, multi-stage framework integrating quantitative proteomics with curated biological and clinical metadata. The architecture was optimized to enable systematic prioritization of cell surface proteins while accommodating dataset-specific variability and uncertainty inherent to large-scale proteomic analyses. The pipeline consists of four core stages: (i) construction of an annotated union dataset, (ii) parallel multi-parameter filtering, (iii) manual curation for extracellular accessibility, and (iv) composite scoring and prioritization (Figure 1).

### 2.3. Annotated Union Dataset Construction

All datasets were merged into a unified, protein-level table using standardized protein identifiers. For each protein, the annotated union table included quantitative protein expression ranks and differential expression metrics derived from CPTAC COAD and COADREAD cohorts, subcellular localization annotations obtained from UniProt, Human Protein Atlas (HPA), and the Cell Surface Protein Atlas (CSPA), normal tissue expression levels from HPA and GTEx, expression in colorectal cancer cell lines from the Cancer Cell Line Encyclopedia (DepMap), and clinical development metadata from Clarivate Drug Discovery Intelligence (CDDI) and SwissProt.

To systematically assess therapeutic accessibility, a custom localization score was generated for each protein based on curated annotations indicating plasma membrane localization, transmembrane topology, and presence of extracellular domains. Secreted proteins and proteins exclusively annotated to intracellular compartments were flagged for exclusion in later steps.

### 2.4. Parallel Filtering Strategies

Rather than applying a single optimal filtering scheme, the pipeline implements multiple complementary filtering strategies in parallel (Table 1, Table 2 and Table 3). Each strategy represents a distinct combination of selection criteria targeting overexpression in metastatic disease, plasma membrane localization, minimal normal tissue expression, and expression in cancer cell lines. This design reduces dependence on any single parameter threshold and allows identification of candidates that are robust to methodological variability.

Filtering strategies were grouped conceptually into three categories: (i) rank-based strategies combining protein abundance, localization scores, and normal tissue penalties; (ii) differential expression-based strategies focused on upregulated plasma membrane proteins; and (iii) cohort-driven strategies applying stricter differential expression cut-offs within individual CPTAC cohorts. All strategies shared core architectural principles but differed in stringency and parameter weighting, enabling broad exploration of the target space while preserving biological relevance.

Each filtering strategy generated an independent shortlist of candidate proteins. Proteins detected across multiple strategies were considered more robust, reflecting consistency across analytical perspectives. To quantify cross-strategy recurrence, each candidate was assigned a weighted strategy support score. Candidates identified by the broader, less stringent filtering strategies 1–3 and 11 were assigned a weight of 1, whereas candidates identified by the more stringent strategies 4–10 were assigned a weight of 2. Strategies 4–10 were considered higher stringency because they applied stricter localization, expression, differential-expression, normal tissue, or manual curation criteria. The weighted score was used only as a robustness metric for comparing candidate recurrence across filtering strategies and was not used to determine whether a protein passed any individual filter. Candidates with a weighted score greater than 1 were prioritized for visualization and downstream validation because they were supported either by multiple strategies or by at least one high-stringency strategy.

To improve reproducibility, each filtering strategy is summarized in Supplementary Table S1, including its rationale, input datasets, threshold criteria, exclusion criteria, and whether manual curation was applied. The eleven strategies were not intended to represent competing optimized models, but complementary analytical views of the same integrated dataset. Strategies 1–4 emphasized combined rank-based prioritization using protein expression, localization, normal tissue expression, cancer cell line expression, and exclusion of previously explored radiopharmaceutical targets. Strategies 5–7 focused on differential expression of plasma membrane proteins across the COAD and COADREAD cohorts, combined with normal tissue penalties and manual review for extracellular accessibility. Strategies 8–11 applied cohort-driven differential expression criteria to either COAD or COADREAD independently, allowing assessment of candidates that may be sensitive to cohort-specific signals. Candidates recurring across multiple strategies were considered more robust because they were identified under different threshold assumptions and evidence combinations.

### 2.5. Manual Curation for Extracellular Accessibility

Candidates emerging from the automated filtering steps were subjected to manual curation to confirm extracellular accessibility and therapeutic relevance. This step focused on reviewing protein topology, domain structure, and annotation consistency to exclude proteins lacking extracellular epitopes despite partial membrane association. For candidates with uncertain or conflicting localization annotations, additional manual review was performed through focused literature searches and inspection of available source information to confirm evidence of plasma membrane localization and likely extracellular exposure. Manual curation was essential to eliminate false positives arising from incomplete or ambiguous database annotations and to ensure compatibility with antibody-based and radioligand-based therapeutic modalities [1,8].

### 2.6. Composite Scoring and Prioritization Framework

Final prioritization of candidate targets was performed using a composite scoring framework integrating multiple translationally relevant parameters. The composite score was developed as a pragmatic prioritization tool rather than a statistically optimized prediction model. The score incorporated plasma membrane/localization evidence, metastatic colorectal cancer protein expression, clinical/druggability annotation (Supplementary Table S2), cancer cell line expression, and normal tissue expression.

The score was calculated by adding the sum of scaled localization ranks, mean stage IV protein rank, and scaled sum of CDDI ranks, as these parameters represent distinct and relatively independent dimensions of target prioritization: surface accessibility, tumor-associated protein expression, and prior clinical or drug development relevance. To incorporate cancer cell line support, the mean abundance score in all CCLE cell lines and the mean abundance score in CRC-derived CCLE cell lines were averaged before being added to the score. This was done because both parameters derive from related cell line expression datasets, and averaging minimized over-weighting of this evidence layer. In contrast, mean abundance scores from HPA and GTEx were subtracted individually rather than averaged, because normal tissue expression represents a critical safety-related consideration for surface-directed therapeutic development and potential on-target/off-tumor toxicity.

Thus, the composite score was calculated as: Composite score = scaled localization rank + mean stage IV protein rank + scaled CDDI rank + average CCLE expression scores − HPA mean abundance score − GTEx mean abundance score.

Higher scores indicate stronger prioritization based on tumor-associated expression, surface localization, cancer cell line support, and translational evidence, while penalizing broad normal tissue expression. The score is intended to guide selection of candidates for downstream validation and should not be interpreted as definitive evidence of therapeutic suitability.

### 2.7. Validation Layers

The validation layers were designed to address distinct translational risks associated with cell surface target discovery. Protein expression analyses were used to confirm that candidate targets were detectable and enriched in relevant colorectal cancer samples, particularly advanced-stage disease. Subcellular localization and immunohistochemistry data were used to assess whether candidate proteins were likely to be accessible to surface-directed therapeutic or imaging modalities. Transcriptomic and cancer cell line analyses provided orthogonal support for candidate expression and helped identify relevant experimental model systems for downstream validation. Literature and biomarker analyses were used to determine whether candidates had prior evidence of biological, prognostic, diagnostic, or therapeutic relevance. Finally, cross-cancer expression analyses were included to assess whether candidate targets showed broader oncologic relevance or more restricted CRC-associated expression. Together, these validation layers provide supportive evidence for prioritization, while definitive therapeutic suitability will require dedicated experimental studies evaluating epitope accessibility, binding, internalization, biodistribution, and therapeutic index.

To contextualize and illustrate the performance of the multi-layered target discovery pipeline, selected high-ranking candidate proteins were evaluated using several independent validation layers. These analyses were not intended as definitive experimental validation, but rather as orthogonal assessments addressing key translational risks, including expression robustness, localization accuracy, relevance beyond the discovery dataset, and prior clinical or biomarker evidence.

### 2.8. Protein Expression Visualization

Quantitative protein expression patterns were visualized across tumor stages using swarm plots of normalized protein abundance from the CPTAC COAD and COADREAD cohorts. These plots enabled assessment of stage-associated expression trends, inter-patient variability, and consistency between cohorts, with particular emphasis on overexpression in advanced and metastatic disease. Normal tissue reference samples were included where available to contextualize tumor-specific expression.

### 2.9. Transcriptomic Analysis

To assess concordance between protein-level signals and transcriptomic data, mRNA expression patterns for candidate targets were examined in colorectal cancer cell lines from the Cancer Cell Line Encyclopedia (DepMap) and patient tumor samples from The Cancer Genome Atlas (TCGA). Heatmaps were generated to visualize relative expression across samples and to evaluate whether proteomic signals were supported by transcriptional data, recognizing that discordance between RNA and protein expression may occur for membrane-associated proteins.

### 2.10. Immunohistochemistry and Subcellular Localization

Protein localization and tissue-level expression patterns were examined using immunohistochemistry data from the Human Protein Atlas (HPA). Emphasis was placed on confirming plasma membrane staining and differential expression between colorectal cancer tissue and normal colon, where available. This layer served as an independent qualitative assessment of extracellular accessibility and supported exclusion of candidates with ambiguous or predominantly intracellular localization.

### 2.11. Literature Mining and Biomarker Evidence

To evaluate prior evidence linking candidate targets to cancer biology and clinical investigation, systematic literature mining was performed using PubMed. The number of publications reporting associations with cancer, metastatic disease, and metastatic colorectal cancer was recorded. This analysis provided contextual insight into existing biological and clinical knowledge, without presupposing target validity or drugability.

### 2.12. Protein Expression in Other Cancer Types

To assess whether candidate targets exhibited broader oncologic relevance or tumor-type specificity, protein expression data from additional CPTAC cohorts were examined, including breast invasive carcinoma (BRCA; PDC000120), lung squamous cell carcinoma (LSCC; PDC000234), lung adenocarcinoma (LUAD; PDC000153), ovarian serous cystadenocarcinoma (OV; PDC000110), and pancreatic ductal adenocarcinoma (PDAC; PDC000270). Comparative analysis across indications helped contextualize colorectal cancer findings and informed prioritization based on potential therapeutic breadth or selectivity.

### 2.13. Clinical and Biomarker Annotation

Finally, candidate targets were evaluated using curated clinical metadata from Clarivate Drug Discovery Intelligence (CDDI) to determine whether they had previously been reported as biomarkers or explored in therapeutic development. Metrics included number of reported indications, cancer-specific usage, and highest development phase where applicable. This layer provided translational context and assisted in distinguishing well-characterized targets from emerging or underexplored candidates.

Collectively, these validation layers provide complementary evidence for target prioritization by addressing expression robustness, surface accessibility, biological relevance, clinical context, and potential therapeutic breadth. However, they should be interpreted as supportive validation only, and functional therapeutic suitability will require dedicated experimental studies. Localization and accessibility assessments were particularly emphasized due to their importance for radiopharmaceutical and antibody-based targeting.

## 3. Results

### 3.1. Pipeline Performance Across Filtering Strategies

The multi-layered filtering pipeline was applied to proteomic and metadata from the CPTAC COAD and COADREAD cohorts [6,7] to identify cell surface proteins associated with metastatic colorectal cancer (mCRC) according to the workflow described in Figure 1. Eleven distinct filtering strategies were implemented, each varying in stringency for protein expression, localization, normal tissue expression and expression on cancer cell lines (Table 1, Table 2 and Table 3).

**Figure 2:**
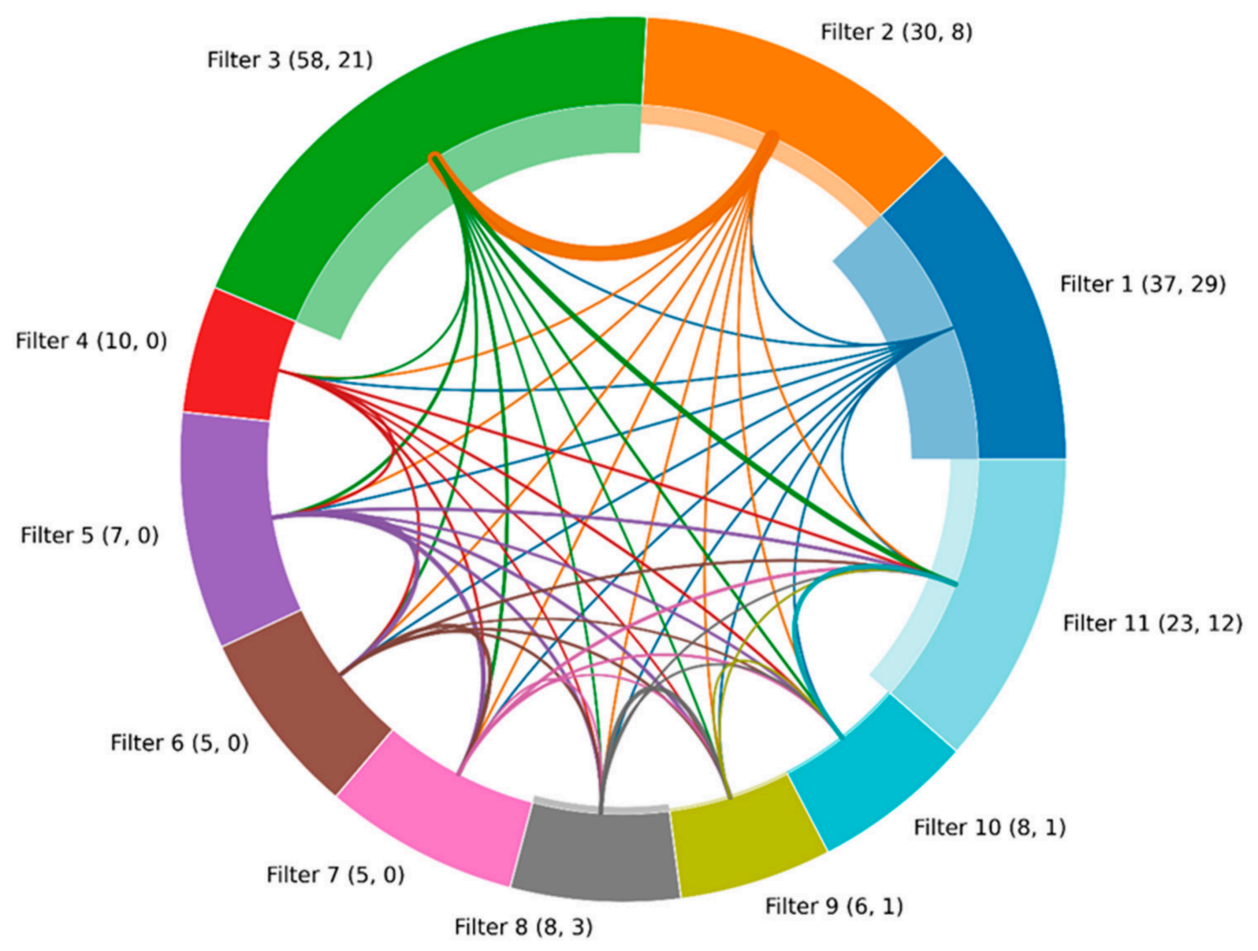
Number of candidate hits per filtering strategy, unique candidates, and overlap between strategies. The chord diagram visualizes pairwise overlap in candidate proteins across eleven filtering strategies used in the multi-layered pipeline. Each arc represents one filtering strategy, with arc length proportional to the total number of retained candidate proteins. Chords connecting strategies indicate shared proteins, with chord thickness reflecting the extent of overlap and chord color corresponding to the originating filtering strategy. Proteins uniquely retained by a given strategy are depicted as an inner band adjacent to each arc, with band thickness proportional to the number of unique candidate proteins for that strategy. The numbers in parentheses after the filter indexes show the total number and the unique number of hits for each filter. Strategies 1–4, based on combined protein ranking, subcellular localization scores, and penalties for normal tissue expression, show limited mutual overlap with later filters. Strategies 5–7, which incorporate differential expression and manual curation for plasma membrane localization, display intermediate connectivity. Strategies 8–11, derived from cohort-driven analyses with increasingly stringent log fold-change thresholds, show increased internal overlap, reflecting shared candidate prioritization under higher stringency. Candidates detected across multiple strategies are considered more robust and are prioritized for downstream validation, while unique candidates highlight potential strategy-specific discoveries.

The number of candidate proteins identified per strategy ranged from 5 to 58 and the number of unique proteins identified for each strategy ranged from 0 to 29, reflecting differences in parameter thresholds and dataset-specific variability (Figure 2). Strategies 1–4, which combined protein rank and localization scores with normal tissue penalties, yielded between 10 and 58 candidates per iteration. Strategies 5–7, incorporating differential expression and manual curation for plasma membrane localization, produced 5–7 candidates. Cohort-driven strategies (8–11), which applied stricter log fold-change cut-offs to either COAD or COADREAD independently, identified 6–23 candidates after manual review.

Candidate targets are identified through multiple complementary filtering strategies applied in parallel, each combining criteria related to metastatic overexpression, plasma membrane localization, extracellular accessibility, and limited normal tissue expression. Manual curation is subsequently performed to confirm extracellular exposure and to exclude proteins with ambiguous or non-therapeutically accessible localization. Final prioritization is achieved using a composite scoring framework that integrates expression, localization, safety related penalties, and translational relevance. Candidates supported by multiple filtering strategies are considered more robust and prioritized for downstream validation.

CPTAC = Clinical Proteomic Tumor Analysis Consortium; COAD = Colon Adenocarcinoma; COADREAD = Colon Adenocarcinoma and Rectal Adenocarincoma; CRC = Colorectal Cancer; BRCA = Breast Invasive Carcinoma; LSCC = Lung squamous cell carcinoma; LUAD = Lung Adenocarcinoma; OV = Ovarian Serous Cystadenocarcinoma; PDAC = Pancreatic Ductal Adenocarcinoma; CDDI = Cortellis Drug Discovery Intelligence; HPA = Human Protein Atlas; GTEx = The Genotype-Tissue Expression project; CSPA = Cell Surface Protein Atlas; CCLE = Cancer Cell Line Encyclopedia; GO = Gene Ontology; TCGA = The Cancer Genome Atlas.

### 3.2. Overlap and Robustness of Candidate Selection

**Figure 3:**
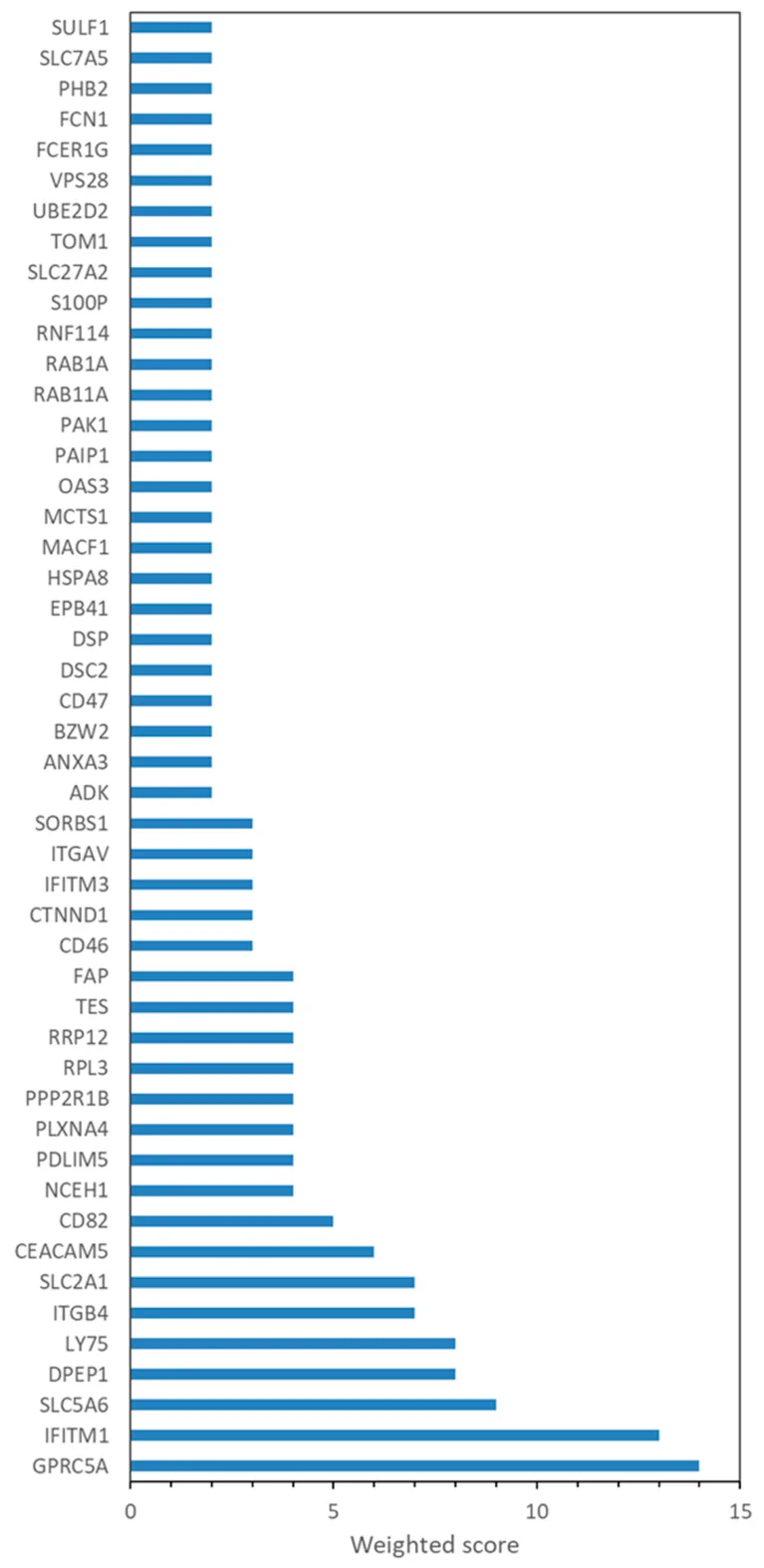
Candidate proteins supported by multiple or high-stringency filtering strategies. Bars show candidate proteins (*N* = 49) with a weighted score greater than 1. The score reflects recurrence across filtering strategies, with higher weights assigned to candidates detected by more stringent strategies as described in the Methods. Higher scores indicate broader or stronger support across the prioritization framework.

To assess robustness, candidate lists from all strategies were compared. A union analysis revealed that only a subset of 43 proteins was identified by more than one strategy. A weighted scoring was implemented where candidates identified by the most stringent filtering strategies (4–10) were assigned a weight of 2 and candidates identified by less stringent strategies (1–3 and 11) were assigned a weight of 1. Figure 3 shows the resulting 48 proteins with a score of more than 1. GPRC5A emerged as the most robust candidate, appearing in 8 different strategies and all major strategy groups (2–4, 5–7, and 8–9). Other recurrent hits included IFITM1, SLC5A6, DPEP1 and LY75, each detected in 5 or more independent strategies (Figure 3).

The average pairwise overlap between filtering strategies was 0.11, indicating limited redundancy between individual approaches. The relatively low average pairwise overlap indicates that the filtering strategies captured complementary rather than redundant candidate sets. This was expected because the strategies were designed to emphasize different biological and translational dimensions, including protein abundance, metastatic-stage expression, plasma membrane localization, normal tissue expression, cancer cell line expression, and cohort-specific differential expression. Thus, low overlap should not necessarily be interpreted as instability alone, but rather as evidence that different filters interrogate distinct regions of the target discovery space. However, limited overlap may also reflect dataset-specific variability and sensitivity to threshold selection. For this reason, recurrence across multiple independent strategies was used as a robustness criterion, with multi-strategy candidates prioritized for downstream evaluation, consistent with the general principle that repeated selection across complementary feature selection procedures can improve confidence in high-dimensional biomarker prioritization [16].

Despite this low overlap, candidates detected across multiple strategies were enriched for known colorectal cancer biomarkers. Specifically, FAP, CEACAM5, ITGAV, and ITGB4 were exclusively identified among multi-strategy candidates (4/39, 10.3%) and were absent among single-strategy hits (0/95), representing a significant enrichment (Fisher’s exact test, *p* = 0.0064). This analysis provides an internal quantitative benchmark supporting cross-strategy recurrence as a marker of biological and translational relevance. However, it should be interpreted as a plausibility assessment rather than definitive external benchmarking against independent target discovery methods. The goal for this analysis was to find unique targets for radiopharmaceuticals. Strategy 5–11 did not contain criteria for exclusion of targets that were already exploited in this field. Therefore, a new round of selection was performed on the data in Figure 3 to remove the candidates where we could find that it had been used as a target for a therapeutic radiopharmaceutical agent. This analysis resulted in exclusion of ITGB4 [17], CEACAM5 [18], Bioorg Med Chem Lett, 2024, FAP [19], CD46 [20] and SLC7A5 [21]. Supplementary Figure S1 shows the resulting bar chart. Importantly, this exclusion was performed as a secondary analysis step and was not incorporated into the core pipeline, ensuring that initial target identification remained unbiased.

### 3.3. Composite Scoring and Prioritization

Final candidate ranking was based on a composite score integrating mean stage IV protein rank, localization score, normal tissue expression penalty, and protein expression in cancer cell lines. Table 4 shows the resulting Top-10 candidates. GPRC5A achieved the highest composite score (4.5), followed by SLC2A1 (4.0), CD47 (2.5), DPEP1 (1.9) and IFITM1 (1.9). These candidates represent diverse protein classes, including a G-protein coupled receptor, a solute carriers, an immune checkpoint regulator, an enzyme and an antiviral effector, highlighting the pipeline’s capacity to capture biologically distinct targets.

GPRC5A was prioritized as the leading candidate because it combined several favorable features across the framework. It was recurrently identified across independent filtering strategies, showed strong support from localization-based scoring, retained expression evidence in metastatic colorectal cancer datasets, and achieved a relatively favorable balance between tumor-associated expression and normal tissue expression penalties. Thus, its high ranking was not driven by a single parameter, but by convergence across expression, localization, and translational scoring layers. This cross-strategy recurrence supports GPRC5A as a robust candidate for further evaluation rather than a strategy-specific hit.

To assess whether the final ranking was dependent on the specific weighting of individual score components, we performed a sensitivity analysis of the top 20 candidates using three alternative weighting schemes (Supplementary Table S3). The ranking was highly stable across models. GPRC5A, SLC2A1/GLUT1, and CD47 remained the three highest-ranked candidates in all models, and the baseline top 10 was largely preserved. Top-10 overlap with the baseline ranking was 9/10 for Model A, 10/10 for Model B, and 9/10 for Model C. These findings indicate that the final prioritization was not driven by a single scoring component and support the robustness of the composite ranking.

### 3.4. Validation

Each validation layer contributed complementary information to candidate interpretation. Expression analyses assessed whether targets were present in relevant tumor samples; localization and immunohistochemistry analyses evaluated surface accessibility; transcriptomic and cell line data provided orthogonal support and model system context; literature and biomarker analyses assessed prior biological and clinical relevance; and cross-cancer analyses helped determine whether candidates may have broader applicability beyond colorectal cancer. These analyses were intended to support prioritization rather than replace experimental validation.

The top 20 candidates were compared with two independent proteogenomic target discovery resources [4,9]. In the Wang et al. tumor-enriched cell surface antigen dataset, three candidates overlapped with the COAD-specific list: GPRC5A, DPEP1, and RRP12 (Supplementary Table S4). When all Wang cancer types were considered, 10 of the top 20 candidates and 9 of the top 10 candidates were represented. Comparison with the Savage/LinkedOmics pan-cancer therapeutic target resource showed that 19 of the 20 candidates had a score above zero, and nine showed increased tumor expression in COAD (Supplementary Table S5). These findings provide independent support for several prioritized candidates while also highlighting that external resources differ in scope, target definitions, and cancer-type coverage.

**Figure 4:**
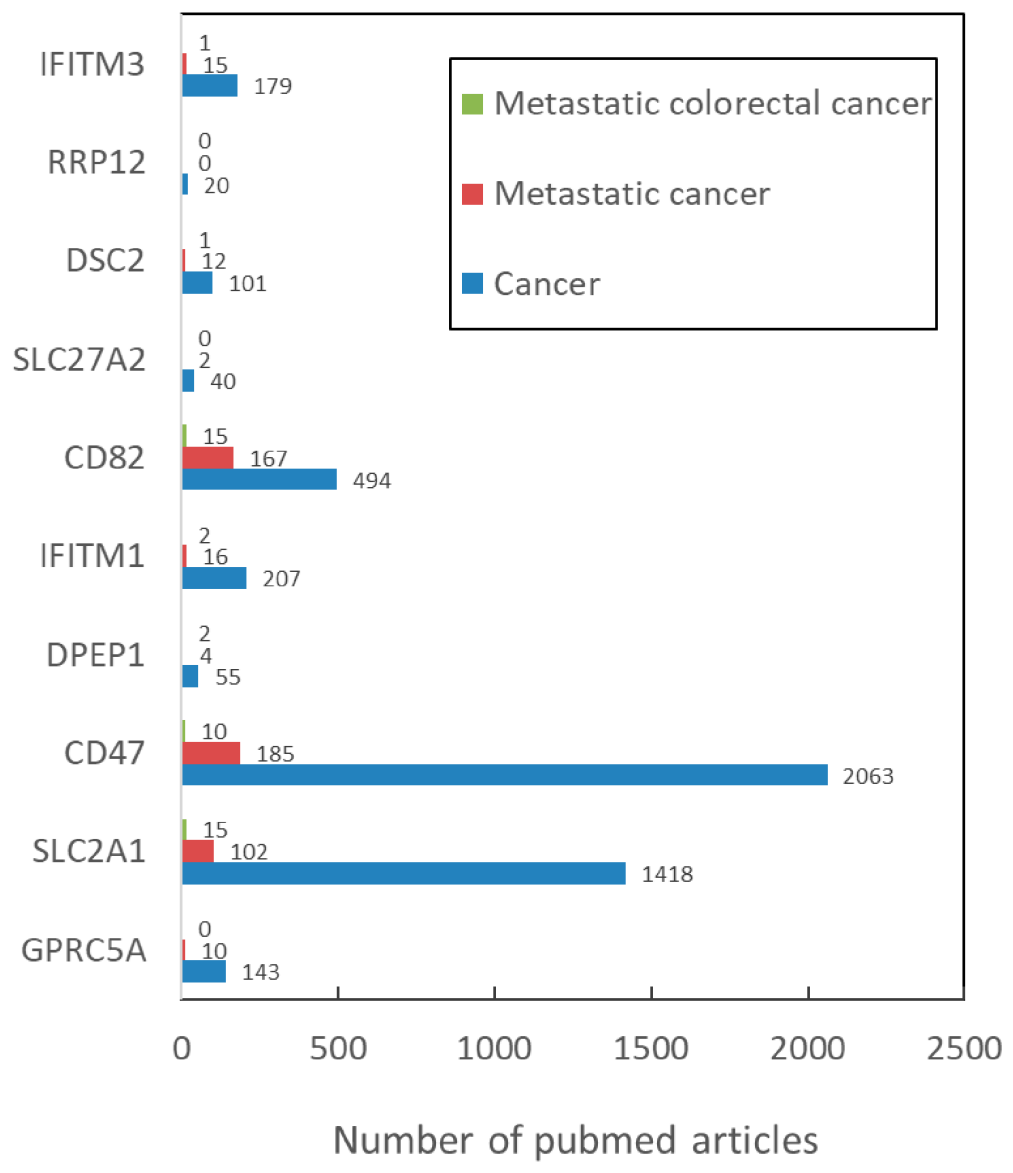
Literature-based contextual validation of top-ranked candidates. PubMed citation counts are shown for searches combining each candidate target with cancer, metastatic cancer, or metastatic colorectal cancer. Citation counts were used as contextual evidence of prior biological or clinical relevance and should not be interpreted as evidence of therapeutic suitability.

To illustrate the translational potential of the pipeline, selected candidates were evaluated using multiple complementary validation layers. PubMed citation analysis showed that all top-ranked candidates have previously been reported in cancer-related contexts, with most also described in metastatic disease, supporting their relevance as established or emerging biomarkers (Figure 4) [22].

The five highest-ranked candidates represent biologically distinct classes of cell surface or membrane-associated proteins, each with different translational opportunities and limitations. To improve clinical interpretability of the ranked candidate list, we summarized the translational characteristics of the five leading candidates in Table 5.

GPRC5A, the top-ranked candidate, is a class C orphan G-protein-coupled receptor that has been associated with tumor progression and adverse outcome in several epithelial malignancies [22]. In colorectal cancer specifically, GPRC5A expression has been reported to promote tumor growth and invasion through non-coding RNA-mediated regulatory mechanisms [23]. Its recurrent identification across multiple filtering strategies, together with favorable localization and expression features, supports its prioritization as a candidate for further evaluation in surface-directed imaging or therapeutic development.

SLC2A1 encodes GLUT1, a glucose transporter frequently linked to tumor metabolism and increased glycolytic activity. Although its plasma membrane localization and cancer-associated expression make it biologically attractive, expression in metabolically active normal tissues may limit its therapeutic window and would require careful evaluation of toxicity and target selectivity [24,25].

CD47 is a well-established innate immune checkpoint that inhibits macrophage-mediated phagocytosis through interaction with SIRPα [26,27]. Its recovery among the top-ranked candidates supports the ability of the pipeline to identify clinically relevant cell surface targets, although widespread expression on normal hematopoietic cells represents an important limitation for direct targeting.

DPEP1 is a GPI-anchored membrane enzyme previously associated with colorectal cancer progression, invasion, and metastasis, and its surface anchoring may make it suitable for antibody- or ligand-based approaches [29,30].

IFITM1 is an interferon-induced transmembrane protein implicated in colorectal cancer invasion and metastatic behavior; its membrane association and previous investigation as an imaging or targeting antigen suggest potential translational relevance, although further validation of extracellular accessibility and tumor specificity is required [32,33].

**Figure 5:**
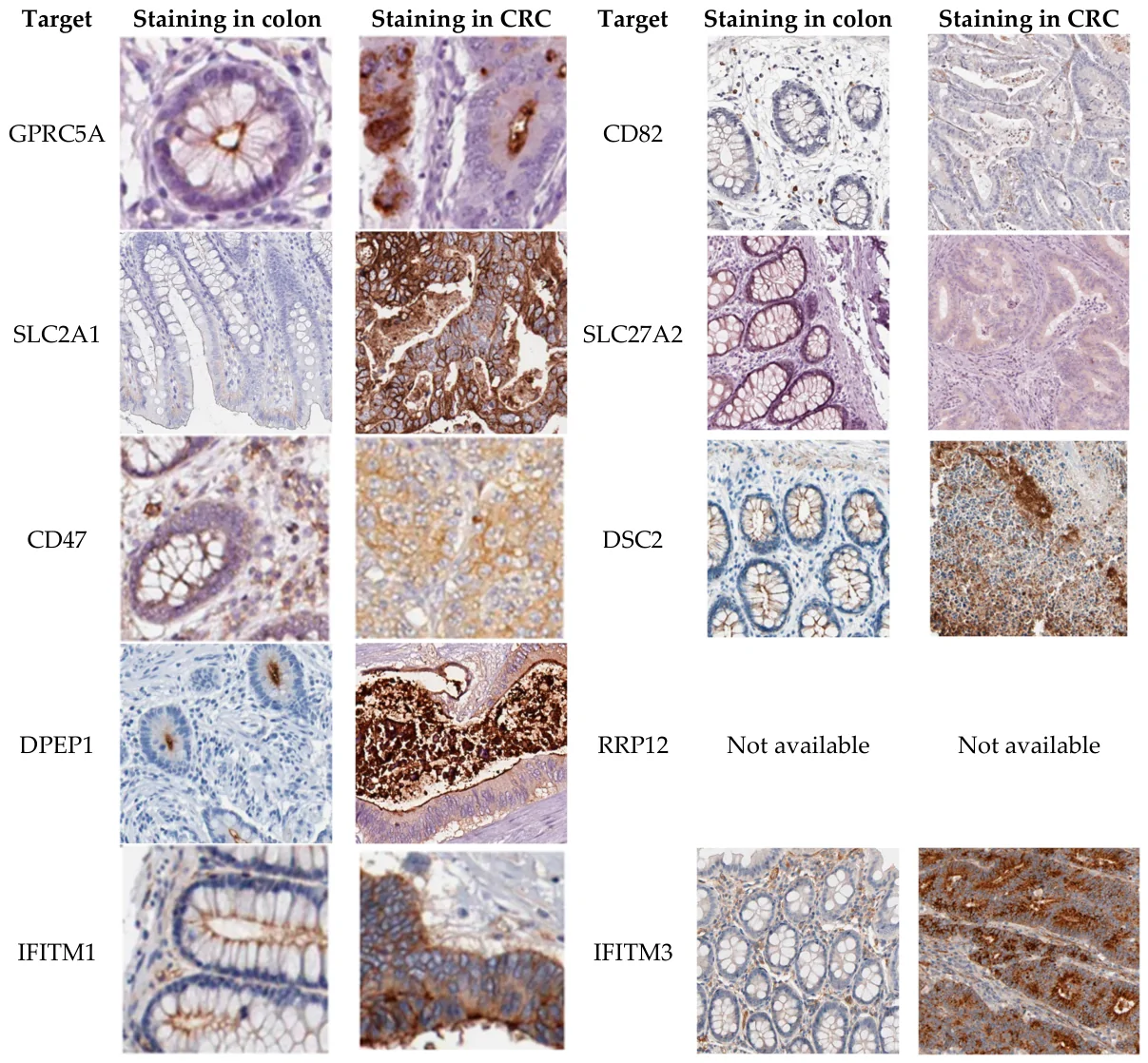
Immunohistochemical validation of selected candidate targets. Representative Human Protein Atlas images (proteinatlas.org [9]) show protein expression in normal colon and colorectal cancer tissue. Brown staining indicates target expression. HPA annotations were used to assess staining intensity and membrane localization where available. Images for RRP12 were not available. Tumor expression for CD82 and SLC27A2 could not be confirmed and IHC images were not available for RRP12. Staining intensity was evaluated semi-quantitatively based on HPA annotation (low, medium, high).

**Figure 6:**
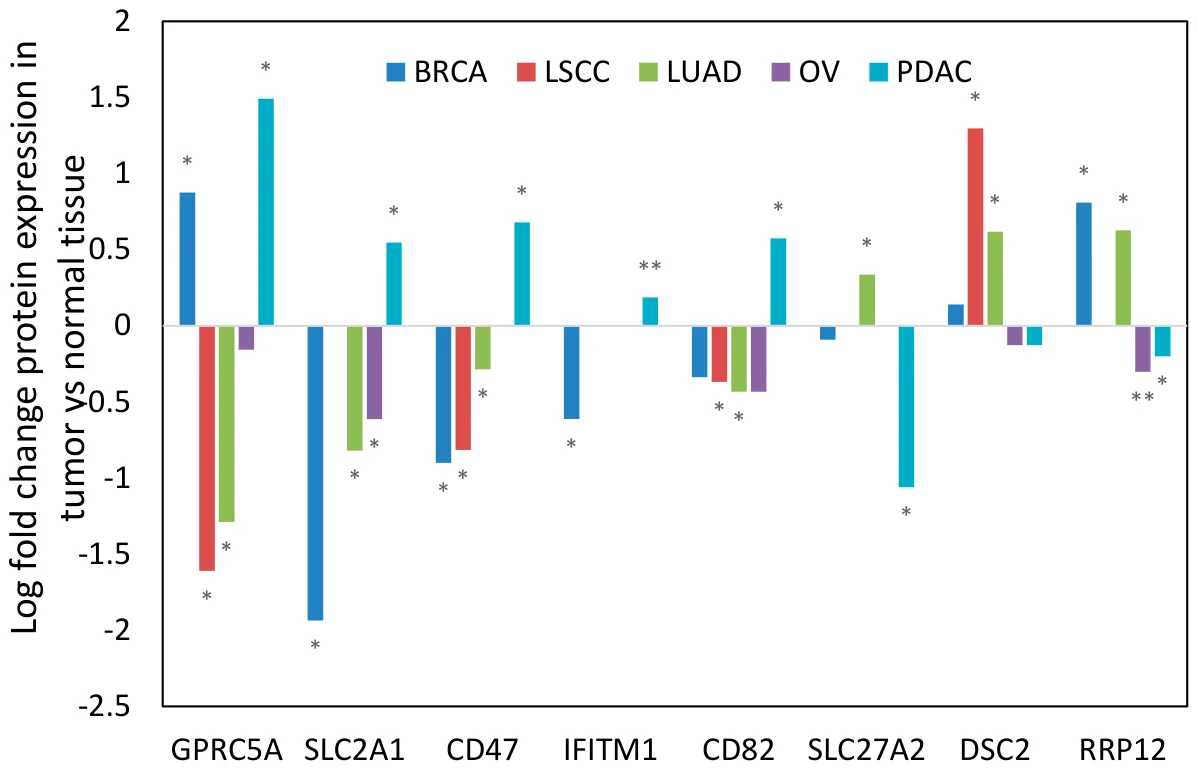
Expression of top-ranked candidates across additional cancer types. Differential protein expression of selected candidates was assessed using CPTAC datasets from breast invasive carcinoma, lung squamous cell carcinoma, lung adenocarcinoma, ovarian serous cystadenocarcinoma, and pancreatic ductal adenocarcinoma. Statistical significance is indicated where available. * adj. *p*-value < 0.001, ** adj. *p*-value < 0.05. Data were not available for DPEP1 and IFITM3.

**Figure 7:**
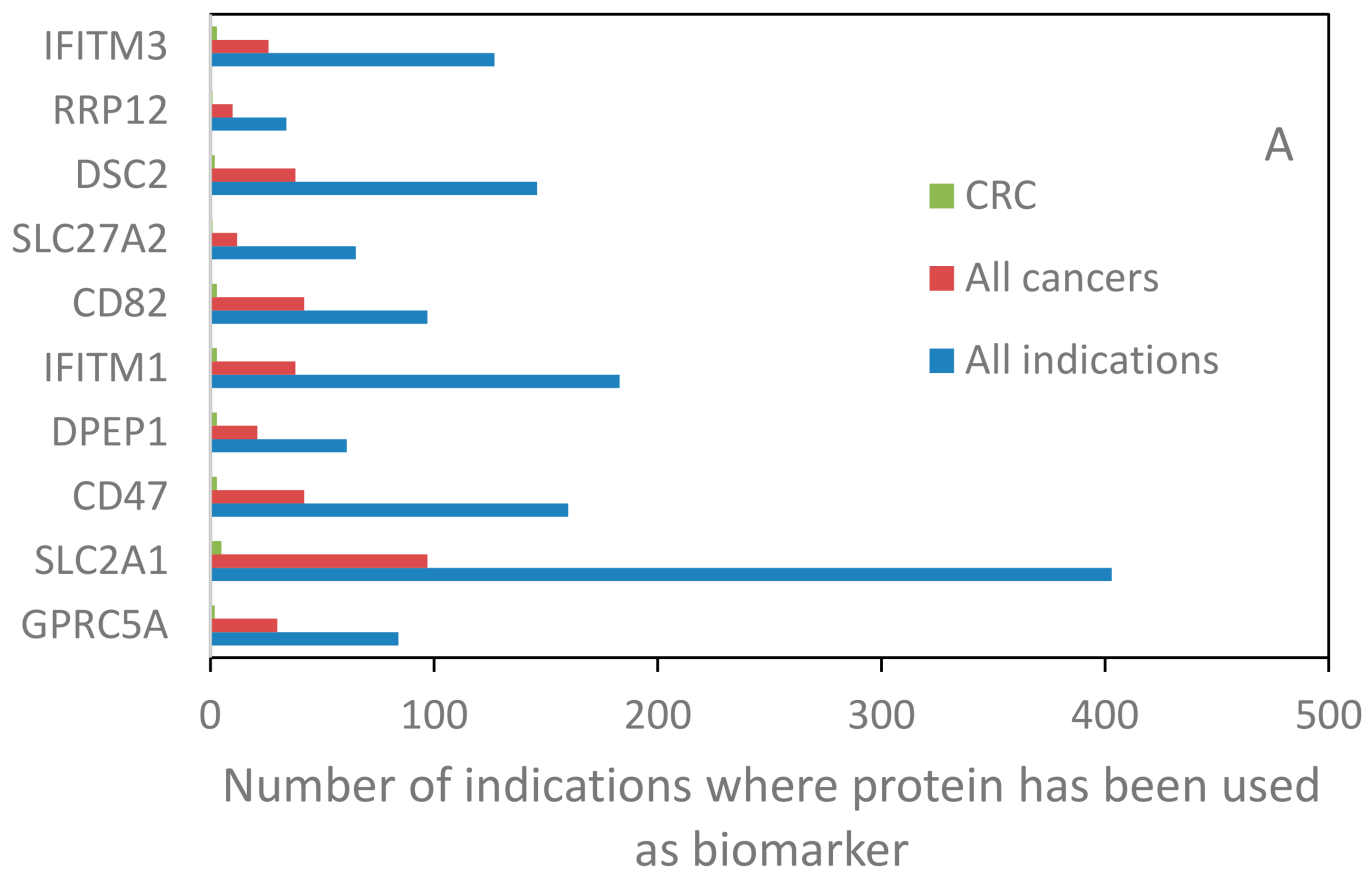

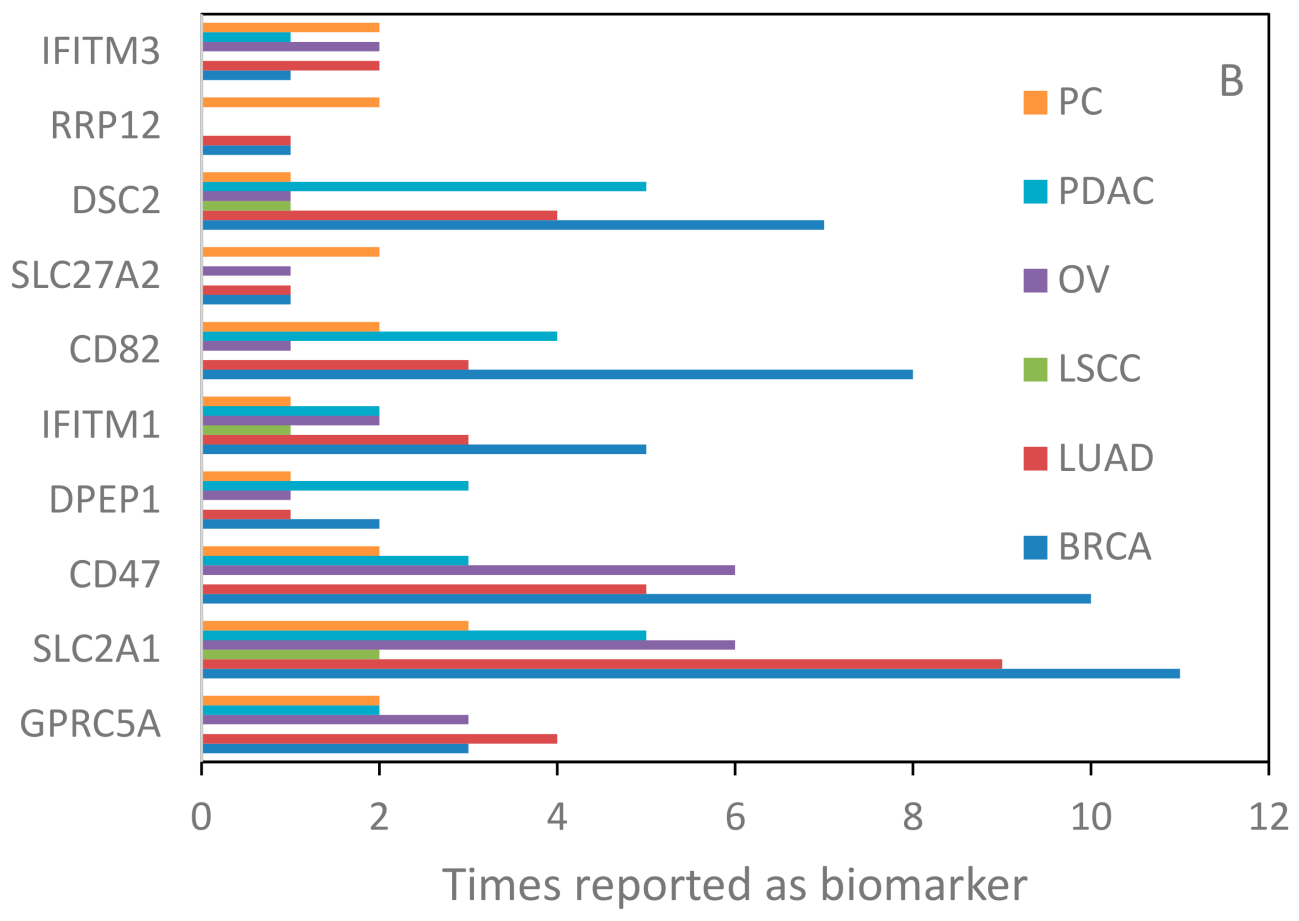
Prior biomarker evidence for top-ranked candidates. The figure summarizes the number of indications and cancer types in which each candidate has been reported as a biomarker according to curated clinical annotation data. These data provide translational context but do not establish therapeutic suitability. (**A**) Number of indications protein has been used as biomarker across indications, cancers, CRC and times used. (**B**) Times reported as biomarker for different tumor types.

Immunohistochemistry data from the Human Protein Atlas further verified plasma membrane localization for GPRC5A, SLC2A, CD47, DPEP1, IFITM1, DSC2 and IFITM3 in colorectal cancer tissues (Figure 5). In addition, protein expression analysis across other tumor types demonstrated that most of the top-10 ranked candidates were differentially expressed in multiple cancer indications (Figure 6). Finally, clinical annotation revealed that all top-ranked candidates have previously been used as biomarkers, including across different cancer types, further supporting their translational relevance (Figure 7).

### 3.5. Summary of Pipeline Robustness

The integration of proteogenomic data with curated metadata and iterative filtering strategies enabled the identification of candidate cell surface targets for mCRC. The recovery of known biomarkers alongside novel candidates demonstrates the biological plausibility and potential adaptability of the approach. The pipeline’s modular design allows adaptation to other solid tumor indications and therapeutic modalities. Overall, robustness was supported at three levels: recurrence across complementary filtering strategies, stability of the composite ranking under alternative weighting schemes, and partial corroboration by independent proteogenomic target discovery resources.

## 4. Discussion

### 4.1. Principal Findings

In this study, we developed a multi-layered proteogenomic framework for prioritizing therapeutically accessible cell surface targets in mCRC. The principal finding is that integration of protein-level expression, plasma membrane localization, normal tissue expression, cancer cell line support, clinical annotation, and recurrence across complementary filtering strategies can identify both established CRC-associated markers and less-characterized candidate targets [1,9,10]. Candidates detected across multiple strategies were enriched for known cancer-associated surface markers, supporting cross-strategy recurrence as a practical robustness metric. Composite scoring further prioritized GPRC5A, SLC2A1, CD47, DPEP1, and IFITM1 as leading candidates for downstream validation. A key feature of the framework is its use of multiple complementary filtering strategies rather than reliance on a single ranking scheme. This design reduced dependence on any individual threshold or dataset-specific assumption and enabled candidates to be assessed from different analytical perspectives. The recovery of known CRC-associated surface markers such as FAP and CEACAM5 supports the biological plausibility of the approach, while identification of less-characterized candidates such as GPRC5A illustrates its potential to move beyond canonical targets.

The low average pairwise overlap between filtering strategies highlights both a strength and a limitation of the framework. On one hand, it demonstrates that the strategies captured complementary evidence combinations rather than redundant candidate sets. On the other hand, it indicates that selection can be sensitive to thresholds, cohort-specific signals, and dataset heterogeneity. We therefore interpreted cross-strategy recurrence as a conservative robustness metric: candidates identified by multiple strategies are less likely to reflect a single filtering assumption, whereas single-strategy candidates should be considered more exploratory and require stronger independent validation [16]. The supplementary sensitivity analysis and external benchmarking further support the robustness of the candidate ranking.

### 4.2. Comparison with Existing Target Discovery Approaches

Compared with existing target discovery approaches, the present framework differs in several ways [1,9,10]. First, it is indication-focused rather than purely pan-cancer, enabling prioritization within the clinical and biological context of mCRC. Second, it explicitly incorporates translational constraints that are critical for surface-directed modalities, including plasma membrane localization, extracellular accessibility, and normal tissue expression. Third, it integrates cancer cell line expression and clinical/druggability annotations to support downstream experimental planning. Finally, it uses multiple parallel filtering strategies rather than a single optimized ranking model, reducing dependence on any individual threshold or dataset-specific assumption. These features make the framework complementary to existing proteogenomic target discovery resources and particularly suited for early-stage prioritization of targets for radioligand therapy, antibody-based therapies, antibody–drug conjugates, molecular imaging, and biomarker development.

Recent pan-cancer proteogenomic atlases have demonstrated the power of large-scale integration of proteomic and genomic data for identifying candidate cancer drug targets, most notably the comprehensive analysis reported by Savage et al. [9]. However, such resources are primarily designed for broad target enumeration, whereas the present framework focuses on indication-specific prioritization of therapeutically accessible cell surface targets. This distinction is particularly relevant for antibody-based therapies and radiopharmaceuticals, where plasma membrane localization and on-target toxicity are dominant determinants of clinical feasibility [1,2].

### 4.3. Clinical and Translational Implications

The translational value of the proposed framework lies in its ability to prioritize cell surface proteins according to criteria directly relevant for clinical development. Candidate targets with favorable tumor-to-normal tissue profiles may first be explored as molecular imaging targets, enabling non-invasive assessment of target expression, lesion heterogeneity, and whole-body biodistribution before therapeutic development. Targets with robust plasma membrane localization and extracellular accessibility may be suitable for radioligand therapy or radioimmunotherapy, provided that sufficient tumor uptake, retention, and dosimetry can be achieved [34]. Proteins with evidence of internalization may be particularly relevant for antibody–drug conjugates or other antibody-based delivery platforms, whereas targets with heterogeneous or context-dependent expression may still have utility as predictive or selection biomarkers [5].

Among the prioritized candidates, GPRC5A is of particular interest because it achieved the highest composite score and was repeatedly identified across independent filtering strategies. Its prioritization was therefore not driven by a single parameter, but by convergence across several layers of evidence, including membrane localization, metastatic colorectal cancer expression, relatively favorable normal tissue expression penalties, and emerging evidence of cancer relevance [22,23]. From a translational perspective, GPRC5A may be attractive for surface-directed imaging or therapeutic development because receptor-like membrane proteins can potentially be targeted using antibodies, ligands, or engineered binding molecules. However, its therapeutic suitability remains unvalidated and requires confirmation of extracellular epitope accessibility, target density, internalization, normal tissue distribution, biodistribution, and therapeutic index. DPEP1 may represent another promising candidate for colorectal cancer-directed targeting because of its GPI-anchored surface localization and reported association with invasion and metastasis. Recent work suggests that DPEP1 may also influence CRC immune biology, with DPEP1 preferentially expressed in MSS CRC and implicated in neutrophil binding and tumor microenvironment organization [31]. In contrast, CD47 and SLC2A1 are biologically and clinically relevant but may pose greater safety challenges because of broader normal tissue expression [24,26,28]. IFITM1 occupies an intermediate position, with evidence supporting cancer-associated expression and membrane localization, but with a need for further experimental confirmation of extracellular accessibility and suitability for therapeutic engagement [32,33]. Thus, the prioritized candidates should be viewed as a ranked set of hypotheses requiring modality-specific validation rather than as equivalent or clinically validated development opportunities.

Tumor heterogeneity represents an important consideration for clinical translation. Metastatic colorectal cancer may show substantial interpatient, interlesional, and intratumoral variability in target expression, which could influence patient selection, imaging sensitivity, and therapeutic efficacy. For surface-directed modalities such as radioligand therapy, antibody–drug conjugates, and antibody-based therapies, heterogeneous target expression may result in incomplete lesion targeting or selection of antigen-low resistant tumor cell populations. Molecular imaging may therefore be valuable as an early translational step, enabling non-invasive assessment of whole-body target expression and lesion-to-lesion variability before target-directed therapy [34].

### 4.4. Limitations

Despite its strengths, the pipeline has limitations. First, it relies on the quality and completeness of public and curated datasets. Differences in cohort composition, tumor purity, sample processing, normalization procedures, and proteomic platform technologies may influence protein detection, abundance estimates, and differential expression results. Such heterogeneity may affect candidate ranking and may partly explain differences between cohort-driven filtering strategies. In addition, resources such as CPTAC, HPA, GTEx, CCLE/DepMap, UniProt, CSPA, and clinical drug-development databases differ in experimental methods, annotation depth, and update frequency, potentially introducing inconsistencies when integrated into a single framework [6,7,9].

Second, the current study focuses on target identification and prioritization and does not include functional validation in vitro or in vivo. Public proteomic, transcriptomic, localization, and biomarker datasets can support target nomination, but they cannot establish whether a candidate protein is therapeutically accessible on viable tumor cells, expressed at sufficient cell surface density, internalized after ligand or antibody binding, retained within tumor lesions, or associated with an acceptable therapeutic index. Consequently, the prioritized candidates should be interpreted as hypotheses for experimental validation rather than as clinically actionable therapeutic targets.

Finally, literature-based metrics and biomarker annotations should be interpreted cautiously. PubMed citation counts and prior biomarker reports may reflect research intensity rather than therapeutic feasibility. Well-studied proteins may appear more strongly supported, whereas less-characterized proteins may be underrepresented despite potentially favorable properties. Conversely, prior biomarker use does not imply suitability for therapeutic targeting. For this reason, literature mining and biomarker annotation were used only as contextual validation layers and not as proof of druggability or clinical utility.

Manual curation also represents a reproducibility limitation. Although manual review improves biological plausibility by helping exclude proteins unlikely to be extracellularly accessible, it may introduce investigator-dependent subjectivity. Future versions of the framework should incorporate standardized curation criteria, predefined annotation hierarchies, and automated protein topology or cell surface localization prediction tools to improve scalability and reproducibility.

### 4.5. Future Directions

Future development of the framework should focus on improving reproducibility, scalability, and biological resolution. Automation of curation steps through protein topology prediction tools, machine learning-based localization annotation, and standardized evidence hierarchies may reduce subjectivity. Integration of additional omics layers, including phosphoproteomics, mutational data, single-cell RNA sequencing, spatial transcriptomics, and spatial proteomics, may further improve target prioritization by linking expression patterns to functional relevance, cellular source, and spatial heterogeneity [9,10]. Single-cell and spatial methods may be particularly useful for distinguishing tumor-cell-intrinsic expression from stromal, endothelial, or immune-cell expression and for determining whether candidate targets are homogeneously expressed within lesions or variable between primary and metastatic sites. Finally, applying the framework to additional solid tumor indications will be important for assessing generalizability and supporting broader use in radioligand therapy, antibody-based therapy, antibody–drug conjugate development, bispecific antibodies, and molecular imaging.

## 5. Conclusions

We developed a multi-layered proteogenomic prioritization framework for the systematic identification of cell surface therapeutic targets, using mCRC as a proof-of-concept indication. The framework integrates quantitative proteomic data with curated information on protein localization, normal tissue expression, cancer cell line expression and clinical or biomarker annotation. By combining eleven complementary filtering strategies with manual curation and composite scoring, the approach incorporates key translational considerations early in target prioritization, including tumor-associated expression, plasma membrane localization, extracellular accessibility, and potential normal tissue liability.

Application of the framework to mCRC identified several candidate targets, including GPRC5A, SLC2A1, CD47, DPEP1 and IFITM1.

The recovery of established colorectal cancer-associated surface markers and the enrichment of known markers among multi-strategy candidates support the biological plausibility of the approach. In addition, cross-strategy recurrence and composite scoring provided complementary prioritization metrics, helping to distinguish candidates supported by multiple evidence layers from more exploratory single-strategy findings.

The identified candidates should be interpreted as prioritized hypotheses for experimental validation rather than as clinically validated therapeutic targets. Further work will be required to confirm tumor-selective cell surface expression, extracellular epitope accessibility, target density, internalization, biodistribution, dosimetry, and therapeutic index. Nevertheless, the modular design of the framework may support early-stage selection of targets for molecular imaging, radioligand therapy, antibody-based therapies, antibody–drug conjugates, and biomarker development. Future refinement should focus on experimental validation, automation of manual curation steps, integration of single-cell and spatial technologies, and validation across additional tumor indications.

## Figures and Tables

**Table 1:** Filtering strategies 1–4 using protein expression, localization and excretion data for biopsies and tumor cells as well as normal cell expression and information about use in RLT.

Filter	Rationale	1	2	3	4
Sum of scaled localization ranks (UniProt, HPA & CSPA)	Ensure localization on PM	>0.44	≥0.1		>0.4
Mean protein rank	Overexpression of protein		≥0.5		
Mean stage 4 protein rank	Proteins with stage 4 protein expr	>0		>0	>0.3
Targeted by RIT	Protein with other RITs removed	=0			
UniProtKB & HPA secreted	No secreted proteins		=0		
GTEx mean abundance	Low normal tissue expr		<2		<1.8
HPA max abundance	Low normal tissue expr	=0.1, 2 *			
HPA mean abundance	Low normal tissue expr	<2 *			<1.8
Max abundance on CCLE	Expressed on tumor cells	>0.46 *	>1 *	>0.5 *	>2 *
Mean abundance on CCLE	Expressed on tumor cells	>0.4 *	>0.5 *	>0.4 *	>1 *
N detected (CRC) in CCLE	Expressed on tumor cells	>0 *			
Mean abundance CCLE (CRC)	Expressed on tumor cells		≥1.5 *	≥1.4 *	≥1.5 *

* Proteins with no data > (blanks) included. Black cells: Filter not used. HPA = Human Protein Atlas; GTEx = The Genotype-Tissue Expression project; CSPA = Cell Surface Protein Atlas; CCLE = Cancer Cell Line Encyclopedia; CRC = Colorectal Cancer.

**Table 2:** Filtering strategies 5–7 centered on differential expression of plasma membrane proteins looking at data from the COAD and COADREAD cohorts in sequence. The derived shortlists were investigated in later comparative analysis to results from strategies 1–4, where a sum score for mean stage 4 protein rank, localization rank, mean abundance normal tissue and clinical development stage was calculated.

Filter	Rationale	5	6	7
Stage IV protein expression in COAD and COADREAD cohorts	Proteins with stage IV protein expression	At least upregulated in one cohort and upregulated or non-differentially expressed in the other *		
UniProt localization score **	Enrichment of candidate proteins with cell surface localization	>0		>0
Sum of scaled localization ranks (UniProt, HPA & CSPA)	Ensure localization on PM		>0.5	>0
Penalty for secreted proteins	Ensure localization on PM	Shedded proteins excluded		
1.6× Upregulation observed at min in COAD cohort	Increasing selection stringency	LogFC COAD ≥ 0.2		
1.6× Upregulation observed at min in one cohort	Increasing selection stringency		logFC(COAD or COADREAD) ≥ 0.2	
Penalty for mitochondria, nucleus and peroxisome localization	Ensure localization on PM	Exclusion of intra-cellular proteins with membrane association		
Mean abundance HPA	Low normal tissue expression	≤1.5		
Manual review for no extracellularly exposed proteins	Ensure relevant PM expression	Manual curation		

* Proteins with no data > (blanks) included. ** Sum of UniProt annotation scores for (1) plasma membrane localization + topologies, (2) Transmembrane domain, (3) Intramembrane domain, (4) Extracellular domain, (5) Cytoplasmic domain. Black cells = the parameter is not used for this filter.

**Table 3:** Filtering strategies 8–11 are driven by either COAD or COADREAD cohorts alone. The derived shortlists were investigated in later comparative analysis to results from strategies 1–4, where a sum score for mean stage 4 protein rank, localization rank, mean abundance normal tissue and clinical development stage was calculated.

Filter	Rationale	8	9	10	11
Stage IV protein expression in COAD	Proteins with stage IV protein expression	Upregulated			
Stage IV protein expression in COADREAD	Proteins with stage IV protein expression			Upregulated	
UniProt localization score **	Enrichment of candidate proteins with cell surface localization	>0		>0	
Manual review for no extracellularly exposed proteins	Ensure relevant PM expression	Manual curation			
Sum of scaled localization ranks (UniProt, HPA & CSPA)	Ensure localization on PM		>0		
Penalty for secreted proteins	Ensure localization on PM			Shedded proteins excluded	
Upregulation observed in COADREAD cohort	Increasing selection stringency			logFC ≥ 2	logFC ≥ 1.4

** Sum of UniProt annotation scores for (1) plasma membrane localization + topologies, (2) Transmembrane domain, (3) Intramembrane domain, (4) Extracellular domain, (5) Cytoplasmic domain. FC = Fold Change.

**Table 4:** Top-10 ranked candidates and composite scores. The composite prioritization score was calculated by adding the sum of scaled localization ranks, mean stage IV protein rank, scaled sum of CDDI ranks. The mean abundance scores in CCLE and CRC-derived CCLE cell lines were averaged before inclusion to avoid over-weighting related cell line expression parameters. Mean abundance scores in HPA and GTEx were subtracted individually to penalize normal tissue expression, reflecting its importance for therapeutic safety. Higher scores indicate stronger prioritization for translational follow-up, but do not represent definitive validation of therapeutic suitability.

Symbol	Description	Sum of Scaled Localization Ranks	Mean Stage 4 Protein Rank	Scaled Sum of CDDI Ranks	MAS * in HPA	MAS * in GTEx	MAS * in CCLE	MAS * (CRC) in CCLE	Composite Prioritization Score
GPRC5A	G protein-coupled receptor class C group 5 member A	1.84	1.00	0.83	0.71	0.44	1.70	2.19	4.47
SLC2A1/GLUT1	Solute carrier family 2 member 1	2.72	0.16	0.92	0.29	1.1	1.54	1.57	3.965
CD47	CD47 molecule	2.32	0.19	0.92	1.22	1.33	1.59	1.56	2.46
DPEP1	Dipeptidase 1	0.36	0.30	0.83	0.42	0.28	0.59	1.67	1.92
IFITM1	Interferon induced transmembrane protein 1	1.5	0.32	0.83	0.78				1.87
CD82	CD82 molecule	1.24	0.51	0.50	0.58	1.31	1.31	1.48	1.76
SLC27A2	Solute carrier family 27 member 2	0.25	0.24	0.00	0.49	0.45	1.49	2.41	1.50
DSC2	Desmocollin 2	1.61	0.23	0.00	1.31	0.97	1.65	2.15	1.46
RRP12	Ribosomal R processing 12 homolog	0.83	0.41	0.00		1.30	1.52	1.52	1.46
IFITM3	Interferon induced transmembrane protein 3	1.00	0.29	0.00		1.14	1.33	1.26	1.45

* MAS: Mean abundance score. Condional formatting of each column with colors are used to improve readability of the table.

**Table 5:** Translational characteristics of selected top-ranked candidate targets.

Target	Cell Surface Accessibility	Main Limitation	Supporting Evidence	Potential Use
GPRC5A	Supported by localization/HPA data.	Requires validation of accessible epitope and biodistribution.	Prognostic/cancer-associated biomarker; implicated in CRC progression biology [22,23].	Imaging or surface-directed therapy candidate.
SLC2A1/GLUT1	Strong plasma membrane localization.	Broad normal expression may limit therapeutic window.	CRC biomarker linked to metabolism and immune infiltration [24,25].	Biomarker/imaging-related target; therapeutic use requires caution.
CD47	Established cell surface immune checkpoint.	Hematologic expression/toxicity risk.	Large CRC profiling and CD47-therapy literature support relevance and limitations [26,27,28].	Immunotherapy comparator/combination target.
DPEP1	GPI-anchored membrane protein.	Normal tissue expression and target density require validation.	CRC biomarker; linked to prognosis, invasion/metastasis, and immune biology [29,30,31].	Imaging, antibody-based, or radioligand candidate.
IFITM1	Membrane-associated.	Extracellular accessibility and inflammation-related expression need validation.	Linked to CRC progression; IFITM1-targeted NIR-II CRC imaging reported [32,33].	Imaging candidate; possible antibody-based targeting.

## Data Availability

The datasets analyzed in this study are publicly available from CPTAC, Human Protein Atlas, GTEx, DepMap/CCLE, UniProt, and the Cell Surface Protein Atlas, as described in Section 2. Derived tables generated during the current study are available from the corresponding author upon reasonable request. Clinical annotation data obtained from Clarivate Drug Discovery Intelligence are subject to database licensing restrictions.

## Supplementary Materials

The following supporting information can be downloaded at: https://www.mdpi.com/article/10.3390/cancers18162570/s1, Figure S1: Candidate proteins (*N* = 43) not used for therapeutic radiopharmaceuticals with a weighted score greater than 1, indicating support from either multiple filtering strategies or at least one high-stringency filtering strategy within the multi-layered prioritization pipeline. The weighted score is calculated as the sum of strategy-specific weights, where candidates identified by stringent filtering strategies (strategies 4–10) are assigned a weight of 2 and candidates identified by less stringent strategies (strategies 1–3 and 11) are assigned a weight of 1. Bars represent the total weighted score for each protein, reflecting the combined strength and breadth of evidence across filtering strategies. By excluding candidates with a weighted score of 1, the visualization focuses on proteins with enhanced robustness, either through cross-strategy recurrence or selection under higher stringency criteria. These candidates are prioritized for downstream analysis and experimental validation; Table S1: Overview of the eleven filtering strategies used for candidate target identification; Table S2: Druggability and clinical efforts for top 10 candidates; Table S3: Sensitivity analysis; Table S4: Comparison with target discovery in Wang et al. 2023 [4]; Table S5: Comparison with target discovery in Savage et al. 2024 [9].

## Abbreviations

The following abbreviations are used in this manuscript:

CPTAC	Clinical Proteomic Tumor Analysis Consortium
COAD	Colon Adenocarcinoma
COADREAD	Colon Adenocarcinoma and Rectal Adenocarcinoma
CRC	Colorectal Cancer
BRCA	Breast Invasive Carcinoma
LSCC	Lung squamous-cell carcinoma
LUAD	Lung Adenocarcinoma
OV	Ovarian Serous Cystadenocarcinoma
PDAC	Pancreatic Ductal Adenocarcinoma
CDDI	Cortellis Drug Discovery Intelligence
HPA	Human Protein Atlas
GTEx	The Genotype-Tissue Expression project
CSPA	Cell Surface Protein Atlas
CCLE	Cancer Cell Line Encyclopedia
GO	Gene Ontology
TCGA	The Cancer Genome Atlas
PC	Prostate Cancer
FC	Fold Change

## References

[1] Di Meo F., Kale B., Koomen J.M., Perna F. (2024). Mapping the cancer surface proteome in search of target antigens for immunotherapy. Mol. Ther..

[2] Crunkhorn S. (2024). Proteogenomics identifies anticancer targets. Nat. Rev. Drug Discov..

[3] Johnson D., Chee C.E., Wong W., Lam R.C.T., Tan I.B.H., Ma B.B.Y. (2024). Current advances in targeted therapy for metastatic colorectal cancer—Clinical translation and future directions. Cancer Treat. Rev..

[4] Wang J., Yu W., D’Anna R., Przybyla A., Wilson M., Sung M., Bullen J., Hurt E., D’Angelo G., Sidders B. (2023). Pan-Cancer Proteomics Analysis to Identify Tumor-Enriched and Highly Expressed Cell Surface Antigens as Potential Targets for Cancer Therapeutics. Mol. Cell. Proteom..

[5] Bosi C., Bartha A., Galbardi B., Notini G., Naldini M.M., Licata L., Viale G., Mariani M., Pistilli B., Ali H.R. (2023). Pan-cancer analysis of antibody-drug conjugate targets and putative predictors of treatment response. Eur. J. Cancer.

[6] Vasaikar S., Huang C., Wang X., Petyuk V.A., Savage S.R., Wen B., Dou Y., Zhang Y., Shi Z., Arshad O.A. (2019). Proteogenomic Analysis of Human Colon Cancer Reveals New Therapeutic Opportunities. Cell.

[7] Zhang B., Wang J., Wang X., Zhu J., Liu Q., Shi Z., Chambers M.C., Zimmerman L.J., Shaddox K.F., Kim S. (2014). Proteogenomic characterization of human colon and rectal cancer. Nature.

[8] Leung K.K., Schaefer K., Lin Z., Yao Z., Wells J.A. (2025). Engineered Proteins and Chemical Tools to Probe the Cell Surface Proteome. Chem. Rev..

[9] Savage S.R., Yi X., Lei J.T., Wen B., Zhao H., Liao Y., Jaehnig E.J., Somes L.K., Shafer P.W., Lee T.D. (2024). Pan-cancer proteogenomics expands the landscape of therapeutic targets. Cell.

[10] Shraim R., Mooney B., Conkrite K.L., Hamilton A.K., Morin G.B., Sorensen P.H., Maris J.M., Diskin S.J., Sacan A. (2025). ImmunoTar-integrative prioritization of cell surface targets for cancer immunotherapy. Bioinformatics.

[11] Navarro-Jimenez M., Gonzalez B., Mulet N., Hierro C., Alonso S. (2025). KRAS-targeted therapies in colorectal cancer: A systematic analysis of mutations, inhibitors, and clinical trials. npj Precis. Oncol..

[12] Uhlen M., Fagerberg L., Hallstrom B.M., Lindskog C., Oksvold P., Mardinoglu A., Sivertsson A., Kampf C., Sjostedt E., Asplund A. (2015). Proteomics. Tissue-based map of the human proteome. Science.

[13] Thul P.J., Akesson L., Wiking M., Mahdessian D., Geladaki A., Ait Blal H., Alm T., Asplund A., Bjork L., Breckels L.M. (2017). A subcellular map of the human proteome. Science.

[14] Barretina J., Caponigro G., Stransky N., Venkatesan K., Margolin A.A., Kim S., Wilson C.J., Lehar J., Kryukov G.V., Sonkin D. (2012). The Cancer Cell Line Encyclopedia enables predictive modelling of anticancer drug sensitivity. Nature.

[15] The UniProt Consortium (2023). UniProt: The Universal Protein Knowledgebase in 2023. Nucleic Acids Res..

[16] Abeel T., Helleputte T., Van de Peer Y., Dupont P., Saeys Y. (2010). Robust biomarker identification for cancer diagnosis with ensemble feature selection methods. Bioinformatics.

[17] Aung W., Tsuji A.B., Sudo H., Sugyo A., Ukai Y., Kouda K., Kurosawa Y., Furukawa T., Saga T., Higashi T. (2017). Combined treatment of pancreatic cancer xenograft with ^90^Y-ITGA6B4-mediated radioimmunotherapy and PI3K/mTOR inhibitor. World J. Gastroenterol..

[18] Tatsumi T., Zhao S., Kasahara A., Aoki M., Nishijima K.I., Ukon N., Kodama T., Takahashi K., Sugiyama A., Washiyama K. (2024). In vivo-stable bis-iminobiotin for targeted radionuclide delivery with the mutant streptavidin. Bioorg. Med. Chem. Lett..

[19] Zboralski D., Hoehne A., Bredenbeck A., Schumann A., Nguyen M., Schneider E., Ungewiss J., Paschke M., Haase C., von Hacht J.L. (2022). Preclinical evaluation of FAP-2286 for fibroblast activation protein targeted radionuclide imaging and therapy. Eur. J. Nucl. Med. Mol. Imaging.

[20] Bidkar A.P., Wang S., Bobba K.N., Chan E., Bidlingmaier S., Egusa E.A., Peter R., Ali U., Meher N., Wadhwa A. (2023). Treatment of Prostate Cancer with CD46-targeted 225Ac Alpha Particle Radioimmunotherapy. Clin. Cancer Res..

[21] Feng S., Hisada K., Yorifuji H., Shirakami Y., Kaneda-Nakashima K. (2025). Astatine-211-Labeled Therapy Targeting Amino Acid Transporters: Overcoming Drug Resistance in Non-Small Cell Lung Cancer. Int. J. Mol. Sci..

[22] Dai L., Jin X., Liu Z. (2021). Prognostic and clinicopathological significance of GPRC5A in various cancers: A systematic review and meta-analysis. PLoS ONE.

[23] He A., Liao F., Lin X. (2025). Circ_0007351 Exerts an Oncogenic Role In Colorectal Cancer Depending on the Modulation of the miR-5195-3p/GPRC5A Cascade. Mol. Biotechnol..

[24] Liu X.S., Yang J.W., Zeng J., Chen X.Q., Gao Y., Kui X.Y., Liu X.Y., Zhang Y., Zhang Y.H., Pei Z.J. (2022). SLC2A1 is a Diagnostic Biomarker Involved in Immune Infiltration of Colorectal Cancer and Associated With m6A Modification and ceRNA. Front. Cell Dev. Biol..

[25] Ren Z., Zhao J., Li S., Yuan H. (2025). Targeting Glucose Transporter 1 (GLUT1) in Cancer: Molecular Mechanisms and Nanomedicine Applications. Int. J. Nanomed..

[26] Arai H., Gandhi N., Battaglin F., Wang J., Algaze S., Jayachandran P., Soni S., Zhang W., Yang Y., Millstein J. (2024). Role of CD47 gene expression in colorectal cancer: A comprehensive molecular profiling study. J. Immunother. Cancer.

[27] Li Q., Vignali P., Tang D., Martinelli G., Fuochi B., Sparavelli R., Poma A.M., Bruno R., Macerola E., Ugolini C. (2025). Expression and Clinical Significance of CD47 in Colorectal Cancer: A Review. Cancers.

[28] Guo X., Fu Y., Baran N., Ma W. (2025). CD47-Targeted Therapy in Cancer Immunotherapy: At a Crossroads of Promise and Challenge. Oncol. Res..

[29] Eisenach P.A., Soeth E., Roder C., Kloppel G., Tepel J., Kalthoff H., Sipos B. (2013). Dipeptidase 1 (DPEP1) is a marker for the transition from low-grade to high-grade intraepithelial neoplasia and an adverse prognostic factor in colorectal cancer. Br. J. Cancer.

[30] Park S.Y., Lee S.J., Cho H.J., Kim T.W., Kim J.T., Kim J.W., Lee C.H., Kim B.Y., Yeom Y.I., Lim J.S. (2016). Dehydropeptidase 1 promotes metastasis through regulation of E-cadherin expression in colon cancer. Oncotarget.

[31] Glass S.E., Bechard M.E., Cao Z., Aramandla R., Zhao P., Ellis S.T., Green E.H., Fisher E.G., Smith R.T., Sievers C.K. (2025). Dipeptidase-1-knockout mice develop invasive tumors with features of microsatellite-unstable colorectal cancer. JCI Insight.

[32] Sari I.N., Yang Y.G., Phi L.T., Kim H., Baek M.J., Jeong D., Kwon H.Y. (2016). Interferon-induced transmembrane protein 1 (IFITM1) is required for the progression of colorectal cancer. Oncotarget.

[33] Jin S., Lin R., Guan S., Jiang J., He H., Wei E., Lv Z., Yang W., Huang J., Zheng S. (2026). IFITM1-targeted NIR-II fluorescence imaging enables visualisation of colorectal cancer and metastatic lymph nodes. J. Transl. Med..

[34] Primac I., Tabury K., Tasdogan A., Baatout S., Herrmann K. (2025). The molecular blueprint of targeted radionuclide therapy. Nat. Rev. Clin. Oncol..

