# Supplementary material for: A Multi-Layered Proteogenomic Framework for the Prioritization of Cell Surface Therapeutic Targets: Proof-of-Concept for Metastatic Colorectal Cancer
Source: Cancers (Basel). 2026 Aug 10;18(16):2570. doi: 10.3390/cancers18162570 (PMC13511108; doi:10.3390/cancers18162570)
Supplement: Supplementary file 1 [file cancers-18-02570-s001.zip › cancers-4377123-supplementary.pdf]

Supplementary material

Supplementary Figure S1

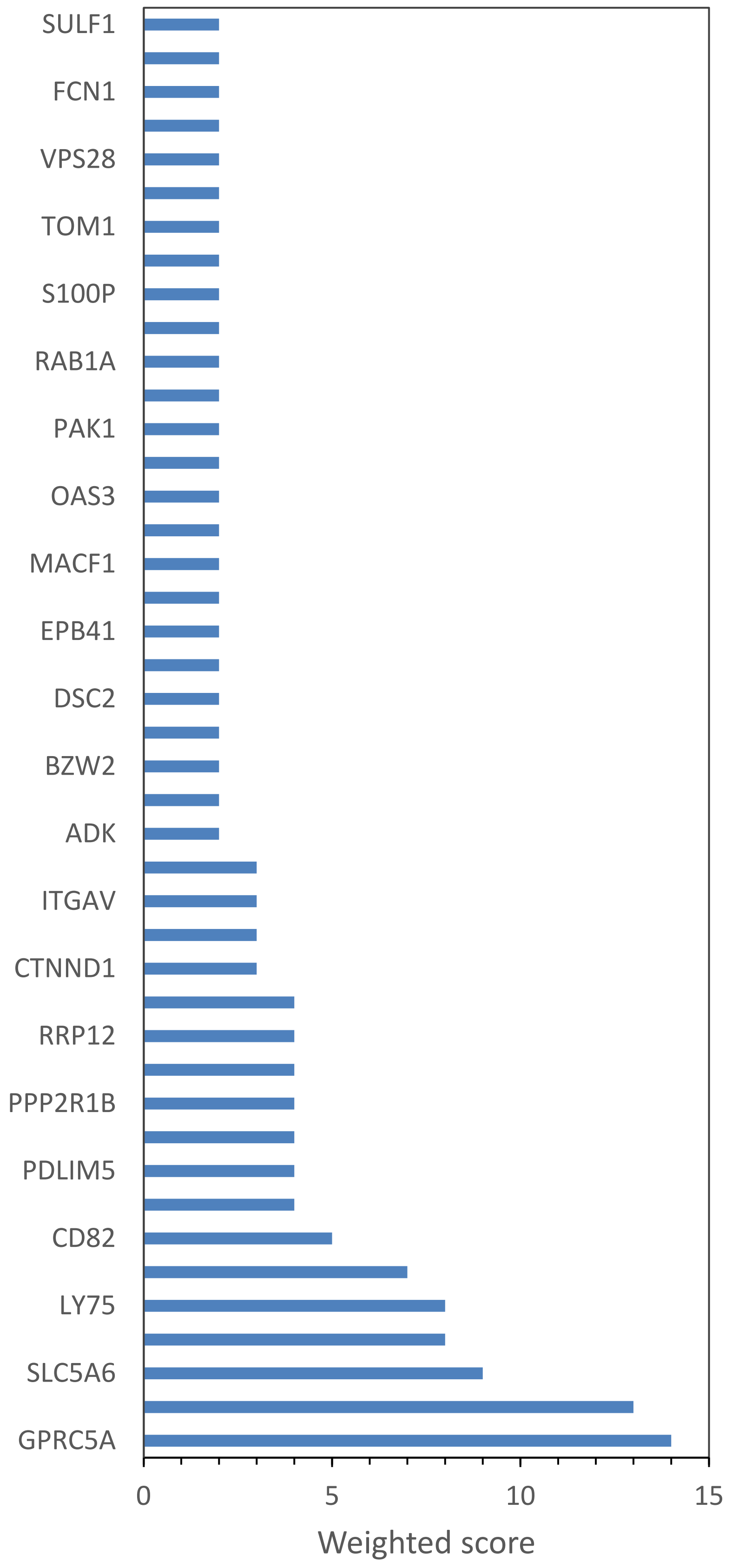

Supplementary Figure S1. Candidate proteins (N=43) not used for therapeutic radiopharmaceuticals with a weighted score greater than 1, indicating support from either multiple filtering strategies or at least one high-stringency filtering strategy within the multi-layered prioritization pipeline. The weighted score is calculated as the sum of strategy-specific weights, where candidates identified by stringent filtering strategies (strategies 4–10) are assigned a weight of 2, and candidates identified by less stringent strategies (strategies 1–3 and 11) are assigned a weight of 1. Bars represent the total weighted score for each protein, reflecting the combined strength and breadth of evidence across filtering strategies. By excluding candidates with a weighted score of 1, the visualization focuses on proteins with enhanced robustness, either through cross-strategy recurrence or selection under higher stringency criteria. These candidates are prioritized for downstream analysis and experimental validation.

Supplementary Table S1. Overview of the eleven filtering strategies used for candidate target identification. Each strategy represents a distinct combination of criteria related to metastatic colorectal cancer protein expression, plasma membrane localization, extracellular accessibility, normal tissue expression, cancer cell-line expression, and prior use in radiopharmaceutical targeting. Strategies 1–4 are primarily rank-based and integrate localization, protein abundance, normal tissue expression, and CCLE expression criteria. Strategies 5–7 are differential-expression-based and incorporate COAD/COADREAD cohort comparisons, localization criteria, normal tissue penalties, exclusion of shedded/secreted or intracellular organelle-associated proteins, and manual curation. Strategies 8–11 are cohort-driven strategies based on either COAD or COADREAD expression signals with additional localization and manual review criteria.

Strategy	Main rationale	Core selection criteria and thresholds	Manual curation
1	Broad rank-based discovery of cell-surface candidates with low normal expression and tumor-cell support	Sum of scaled localization ranks >0.44; mean stage IV protein rank >0; excluded if previously targeted by RIT; HPA max abundance 0–2; HPA mean abundance <2; CCLE max abundance >0.46; CCLE mean abundance >0.4; detected in CRC CCLE cell lines >0	No systematic manual curation at filtering step
2	Rank-based prioritization emphasizing overall protein abundance, localization, low normal expression, and cell-line expression	Sum of scaled localization ranks ≥0.1; mean protein rank ≥0.5; excluded if previously targeted by RIT; secreted proteins excluded; GTEx mean abundance <2; HPA mean abundance <2; CCLE max abundance >1; CCLE mean abundance >0.5; CRC CCLE mean abundance ≥1.5	No systematic manual curation at filtering step
3	Stage IV expression-based prioritization with localization and normal tissue constraints	Sum of scaled localization ranks ≥0.1; mean stage IV protein rank >0; excluded if previously targeted by RIT; secreted proteins excluded; GTEx mean abundance <2; HPA mean abundance <2; CCLE max abundance >0.5; CCLE mean abundance >0.4; CRC CCLE mean abundance ≥1.4	No systematic manual curation at filtering step
4	More stringent rank-based prioritization with stronger localization, metastatic expression, and cell-line expression requirements	Sum of scaled localization ranks >0.4; mean stage IV protein rank >0.3; excluded if previously targeted by RIT; secreted proteins excluded; GTEx mean abundance <1.8; HPA mean abundance <1.8; CCLE max abundance >2; CCLE mean abundance >1; CRC CCLE mean abundance ≥1.5	No systematic manual curation at filtering step
5	Differential-expression-based identification of plasma membrane	Stage IV protein expression upregulated in at least one cohort and upregulated or non-differentially expressed in the other; UniProt localization score >0; logFC COAD ≥0.2; shedded/secreted proteins	Yes

Strategy	Main rationale	Core selection criteria and thresholds	Manual curation
	candidates across COAD and COADREAD	excluded; mitochondrial, nuclear, and peroxisomal proteins excluded; HPA mean abundance ≤1.5	
6	Differential-expression-based strategy using combined localization score and normal tissue constraint	Stage IV protein expression upregulated in at least one cohort and upregulated or non-differentially expressed in the other; sum of scaled localization ranks >0.5; logFC in COAD or COADREAD ≥0.2; shedded/secreted proteins excluded; mitochondrial, nuclear, and peroxisomal proteins excluded; HPA mean abundance ≤1.5	Yes
7	Differential-expression-based strategy with broader localization inclusion	Stage IV protein expression upregulated in at least one cohort and upregulated or non-differentially expressed in the other; UniProt localization score >0; sum of scaled localization ranks >0; logFC in COAD or COADREAD ≥0.2; shedded/secreted proteins excluded; mitochondrial, nuclear, and peroxisomal proteins excluded	Yes
8	COAD-driven identification of upregulated plasma membrane candidates	Stage IV protein expression upregulated in COAD; UniProt localization score >0	Yes
9	COAD-driven strategy using combined localization score	Stage IV protein expression upregulated in COAD; sum of scaled localization ranks >0	Yes
10	High-stringency COADREAD-driven identification of plasma membrane candidates	Stage IV protein expression upregulated in COADREAD; UniProt localization score >0; sum of scaled localization ranks >0; shedded/secreted proteins excluded; COADREAD logFC ≥2	Yes
11	COADREAD-driven strategy with lower differential-expression cutoff	Stage IV protein expression upregulated in COADREAD; UniProt localization score >0; sum of scaled localization ranks >0; COADREAD logFC ≥1.4	Yes

Abbreviations: CCLE, Cancer Cell Line Encyclopedia; COAD, colon adenocarcinoma; COADREAD, colon and rectal adenocarcinoma; CRC, colorectal cancer; GTEx, Genotype-Tissue Expression project; HPA, Human Protein Atlas; logFC, log fold change; RIT, radioimmunotherapy.

Supplementary Table S2. Druggability and clinical efforts for top 10 candidates.

Symbol	Highest phase in cancer	N drugs against the target cancer	N drugs against the target CRC	N indications with drug targeting the protein cancer	N indications with drug targeting the protein CRC
GPRC5A					
SLC2A1	Preclinical	4		1	
CD47	Phase III	40	2	24	2
DPEP1					
IFITM1					
CD82					
SLC27A2	Preclinical	1		1	
DSC2					
RRP12					
IFITM3					

Supplementary Table S3. Sensitivity analysis.

Protein	Composite prioritization score	Baseline rank	Model A	Model A rank	Model B	Model B rank	Model C	Model C rank
GPRC5A	4,47	1	5,385	1	4,88	1	4,965	1
SLC2A1/GLUT1	3,965	2	5,325	2	4,425	2	4,045	2
CD47	2,46	3	3,615	3	2,915	3	2,55	3
DPEP1	1,92	4	2,1	7	2,335	4	2,07	4
IFITM1	1,87	5	2,62	4	2,285	5	2,03	5
CD82	1,76	6	2,375	5	2,005	6	2,01	6
SLC27A2	1,50	7	1,625	11	1,5	7	1,62	9
DSC2	1,46	8	2,265	6	1,46	8	1,575	11
RRP12	1,46	9	1,875	9	1,46	8	1,665	7
IFITM3	1,45	10	1,945	8	1,445	10	1,59	10
S100P	1,36	11	1,44	12	1,355	11	1,66	8
RPL3	1,085	12	1,39	15	1,085	13	1,295	12
RAB11A	1,05	13	1,385	16	1,05	14	1,18	13
EPB41	1,035	14	1,41	13	1,035	15	1,1	15
UBE2D2	0,98	15	1,23	17	0,98	16	1,11	14
SLC6A6	0,83	16	1,4	14	0,83	17	0,83	18
OAS3	0,82	17	1,07	19	0,82	18	0,94	16
NT5E	0,81	18	1,85	10	1,225	12	0,9	17
CTNND1	0,505	19	1,17	18	0,505	19	0,82	19
HSPA8	0,495	20	0,765	20	0,495	20	0,585	20

In the sensitivity analysis, three alternative models were generated by increasing the weight of one score component at a time while keeping all other score components unchanged. Model A increases the contribution of plasma membrane/localization evidence by adding 0.5 × the sum of scaled localization ranks to the baseline score. Model B increases the contribution of clinical/translational evidence by adding 0.5 × the scaled sum of CDDI ranks to the baseline score. Model C increases the contribution of metastatic disease expression evidence by adding 0.5 × the mean Stage IV protein rank to the baseline score. Ranks indicate the position of each protein among the top 20 candidates under each scoring model.

Supplementary Table S4. Comparison with target-discovery in Wang et al. 2023 [4].

Protein	COAD match	COAD rank	COAD iBAQ.mean	COAD logFC	Pan-cancer match	Cancer types
GPRC5A	Yes	13	6,56	1,30	Yes	COAD, PDA, UCEC
SLC2A1	No				Yes	ccRCC, UCEC, HNSCC
CD47	No				Yes	UCEC
DPEP1	Yes	7	6,27	0,72	Yes	COAD
IFITM1	No				No	
CD82	No				Yes	UCEC
SLC27A2	No				Yes	UCEC
DSC2	No				Yes	LSCC, UCEC
RRP12	Yes	27	5,45	0,62	Yes	COAD, LSCC
IFITM3	No				Yes	ccRCC
S100P	No				No	
RPL3	No				No	
RAB11A	No				No	
EPB41	No				No	
UBE2D2	No				No	
SLC6A6	No				Yes	LSCC, UCEC
OAS3	No				No	
NT5E	No				No	
CTNND1	No				No	
HSPA8	No				No	

COAD match denotes presence in the COAD-specific Wang et al. 2023 tumor-enriched predicted cell-surface antigen list [4]. Pan-cancer match denotes presence in any cancer-type sheet in Wang Supplementary Table 2. COAD rank, iBAQ.mean, and logFC are taken from the COAD sheet in Wang Supplementary Table 2.

Supplementary Table S5. Comparison with target-discovery in Savage et al. 2024 [9].

Protein	Pharos family	Target tier	Drug	Antigen	Score	Cancer types with increased tumor expression
GPRC5A	GPCR	T5			4	COAD, HNSCC, PDAC, UCEC
SLC2A1	Transporter	T5		TSA	16	CCRCC, COAD, HNSCC, LSCC, PDAC
CD47	Other	T5			5	CCRCC, HNSCC, OV, PDAC, UCEC
DPEP1	Enzyme	T2	Cilastatin		5	COAD
IFITM1	Other				0	
CD82	Other				7	COAD, PDAC
SLC27A2	Transporter				11	LSCC, LUAD, UCEC
DSC2	Other	T5		TSA	3	LSCC, LUAD
RRP12	Other				14	CCRCC, COAD, HNSCC, LSCC, LUAD, UCEC
IFITM3	Other				11	CCRCC, HNSCC, PDAC
S100P	Other				9	CCRCC, COAD, HNSCC, LSCC, PDAC
RPL3	Other	T1	Omacetaxine; mepesuccinate	TSA	15	CCRCC, COAD, LSCC, LUAD, OV, UCEC
RAB11A	Enzyme				8	
EPB41	Other				2	CCRCC
UBE2D2	Other				6	CCRCC
SLC6A6	Transporter	T4			9	LSCC
OAS3	Enzyme				15	CCRCC, COAD, HNSCC, LSCC, LUAD, OV, PDAC
NT5E	Enzyme	T3	oleclumab; mupadolimab		6	CCRC, PDAC
CTNND1	Other			TSA	11	CCRC, PDAC
HSPA8	Other			TSA	14	COAD, LSCC, LUAD, OV, PDAC

T1: Target of drugs approved for oncology; T2: Target of drugs approved for indications other than oncology; T3: Target of drugs classified as investigational or experimental; T4: Potentially druggable target by small molecules; T5: Cell surface membrane protein; TSA: Tumor Specific Antigen. The Savage/LinkedOmics score represents the number of significant results for a given gene across the target-discovery analyses included in the Savage et al. pan-cancer proteogenomic resource [9]. It is a count-based prioritization

metric reflecting the breadth of supporting evidence across analyses and cancer types, rather than a weighted score or a direct measure of colorectal-cancer specificity, surface accessibility, or radiopharmaceutical suitability.
